# Effects of internal cooling on physical performance, physiological and perceptional parameters when exercising in the heat: A systematic review with meta-analyses

**DOI:** 10.3389/fphys.2023.1125969

**Published:** 2023-04-11

**Authors:** Juliane Heydenreich, Karsten Koehler, Hans Braun, Mareike Grosshauser, Helmut Heseker, Daniel Koenig, Alfonso Lampen, Stephanie Mosler, Andreas Niess, Alexandra Schek, Anja Carlsohn

**Affiliations:** ^1^ Working Group Sports Nutrition of German Nutrition Society, Bonn, Germany; ^2^ Institute of Sports Sciences, Johannes Gutenberg-University of Mainz, Mainz, Germany; ^3^ Department of Sport and Health Sciences, Technical University of Munich, Munich, Germany; ^4^ Manfred Donike Institute for Doping Analysis, Institute of Biochemistry, German Sport University Cologne, Cologne, Germany; ^5^ Olympic Center Rhineland-Palatinate/Saarland, Saarbrücken, Germany; ^6^ Institute of Nutrition, Consumption and Health, University of Paderborn, Paderborn, Germany; ^7^ Division of Sports Medicine, Exercise Physiology and Prevention, Center for Sport Science and University Sports, University of Vienna, Vienna, Austria; ^8^ Risk Assessment Strategies, German Federal Institute for Risk Assessment, Berlin, Germany; ^9^ Olympic Center Stuttgart, Stuttgart, Germany; ^10^ Department of Sports Medicine, University Hospital Tübingen, Tübingen, Germany; ^11^ Editorial Team of the Journal Leistungssport, German Olympic Sports Confederation, Frankfurt, Germany; ^12^ Department of Nutrition and Home Economics, University of Applied Science Hamburg, Hamburg, Germany

**Keywords:** ice, menthol, athlete, perceptional cooling, physical cooling, time trial, sweat rate, core temperature

## Abstract

**Background:** An elevated core temperature (*T*core) increases the risk of performance impairments and heat-related illness. Internal cooling (IC) has the potential to lower *T*core when exercising in the heat. The aim of the review was to systematically analyze the effects of IC on performance, physiological, and perceptional parameters.

**Methods:** A systematic literature search was performed in the PubMed database on 17 December 2021. Intervention studies were included assessing the effects of IC on performance, physiological, or perceptional outcomes. Data extraction and quality assessment were conducted for the included literature. The standardized mean differences (SMD) and 95% Confidence Intervals (CI) were calculated using the inverse-variance method and a random-effects model.

**Results:** 47 intervention studies involving 486 active subjects (13.7% female; mean age 20–42 years) were included in the meta-analysis. IC resulted in significant positive effects on time to exhaustion [SMD (95% CI) 0.40 (0.13; 0.67), *p* < 0.01]. IC significantly reduced *T*core [−0.19 (22120.34; −0.05), *p* < 0.05], sweat rate [−0.20 (−0.34; −0.06), *p* < 0.01], thermal sensation [−0.17 (−0.33; −0.01), *p* < 0.05], whereas no effects were found on skin temperature, blood lactate, and thermal comfort (*p* > 0.05). IC resulted in a *borderline* significant reduction in time trial performance [0.31 (−0.60; −0.02), *p* = 0.06], heart rate [−0.13 (−0.27; 0.01), *p* = 0.06], rate of perceived exertion [−0.16 (−0.31; −0.00), *p* = 0.05] and *borderline* increased mean power output [0.22 (0.00; 0.44), *p* = 0.05].

**Discussion:** IC has the potential to affect endurance performance and selected physiological and perceptional parameters positively. However, its effectiveness depends on the method used and the time point of administration. Future research should confirm the laboratory-based results in the field setting and involve non-endurance activities and female athletes.

**Systematic review registration:** https://www.crd.york.ac.uk/PROSPERO/, identifier: CRD42022336623.

## 1 Introduction

High ambient and radiant temperature, absolute humidity, and factors such as urbanization and heat storage in crowded stadiums may cause “heat stress” in athletes (Bongers et al., 2020). Under these conditions, an athlete’s ability to dissipate the exercise-induced heat production is limited, leading to significantly elevated core temperature and increased risk of performance impairments (Bongers et al., 2020) and heat-related illness (Wendt et al., 2007), including disease (heat edema, heat rash, heat cramps, heat syncope), heat exhaustion, and the most severe form, heat stroke (Howe and Boden, 2007). Typical symptoms of heat exhaustion are dizziness, malaise, nausea, vomiting, or excessive fatigue, and without treatment, the potentially life-threatening heat stroke may develop as core temperature elevates >40°C (Howe and Boden, 2007).

Exertional heat illness has been reported at a rate of 0.47 per 10,000 athlete-exposures among US collegiate athletes (Yeargin et al., 2019), with heat cramps, heat exhaustion, and dehydration being the most prominent types and 8.2% of all cases requiring emergency transport. However, the prevalence of heat-associated diseases might rise since athletes will have to train and compete under more challenging thermal conditions. Besides the fact that global warming will probably lead to an increase in the frequency and length of heat waves, including heat waves occurring in previously temperate environments (McGeehin and Mirabelli, 2001), also major sports events are often organized in extremely hot and/or humid conditions [e.g., Olympic Games in Tokio 2020, Fédération Internationale de Football Association (FIFA) World Cup in Qatar 2022].

To reduce the risk of performance impairments and exercise-induced heat illness when exercising in hot-humid conditions cannot be avoided, athletes need to apply cooling strategies before (*pre*-cooling) or during (*mid*-cooling) exercise to lower core temperature. Cooling applications, in general, can improve exercise performance in hot environments due to reductions in thermal strain and an increased heat storage capacity (Bongers et al., 2017). They can be classified into *external* (i.e., cold-water immersion, ice packs, ice vests) and *internal* (ingestion of ice, cold-water, and menthol) applications. Several systematic reviews and meta-analyses demonstrate a positive effect of cooling on physical performance (Jones et al., 2012; Bongers et al., 2015; Ruddock et al., 2017; Choo et al., 2018; Douzi et al., 2019; Jeffries and Waldron, 2019; Zhang, 2019; Bongers et al., 2020; Keringer et al., 2020; Rodríguez et al., 2020), with external applications such as cold water immersion and ice vests being the most effective strategies for pre- and mid-cooling, respectively (Bongers et al., 2020).

However, not every cooling method providing performance benefits in the laboratory setting is feasible for real-life competition. For example, due to sport-specific regulations, practical considerations, local environmental conditions, high performance costs (even though regulations and conditions would allow for external cooling) it might not be possible to employ external cooling such as cold-water immersion or ice vests (Bongers et al., 2020). In contrast, *internal* cooling applications are more applicable during exercise in a field-based setting and are usually well-tolerated and cheap (Bongers et al., 2017).

There exist two different types of internal cooling: physical and perceptional cooling. The application of physical cooling with a medium of high heat capacity (e.g., ice or cold-water ingestion) might cause a decrease in core temperature and a consequent delay in the onset of thermally induced fatigue (Wegmann et al., 2012). In a recent meta-analysis by Zhang 2019, the ingestion of ice-slurries was associated with moderate performance improvements in hot environments (Zhang, 2019). However, internal heat losses caused by the ingestion of cold fluids might decrease the evaporative potential of the skin (Morris et al., 2016; Jay and Morris, 2018). Therefore, some authors recommend to ingest cold-water/ice-slurry only during exercise in hot, humid, and calm conditions, but not in warm, dry, and windy environments (Jay and Morris, 2018). On the other hand, high-intensity exercise may cause excessive elevations in heat production and sweat rate, and small reductions in sweat rate would only minimally reduce evaporative heat loss, suggesting a net beneficial effect (Bongers et al., 2020). Furthermore, it could be argued that a reduction in sweat rate following the ingestion of cold-water or ice-slurry could prevent dehydration-dependent performance decrements (Murray, 2007).

In contrast, *perceptional* cooling may affect physiological outcomes and performance indirectly by inducing a sensation of cooling (Keringer et al., 2020). The most comment agent is menthol, a cyclic monoterpene alcohol that possesses various biological properties such as antimicrobial, anticancer, anti-inflammatory activities, and well-known cooling characteristics (GPP et al., 2013). Internal menthol application leads to an activation of “transient receptor potential melastatin-8" (TRPM8)-channels causing a reduced thermal sensation and physiological effects similar to “physical cooling” (Zheng, 2013). A recently published study by Han et al., 2020 (Han et al., 2020) showed that intranasal menthol activated several brain regions related to nociceptive and trigeminal processing. However, it remains unclear whether and to what extent this activation has a performance impact. Three systematic reviews assessed the effects of internal and external menthol application on performance in the heat (Douzi et al., 2019; Jeffries and Waldron, 2019; Keringer et al., 2020). According to one review, internal menthol was superior to an external application (Jeffries and Waldron, 2019), whereas another review reported contrary results (Keringer et al., 2020), and one did not show any effect (Douzi et al., 2019).

So far, no systematic review has focused exclusively on the effect of different *internal* cooling methods (ice-/cold-water *and* menthol ingestion). In most of the above-mentioned systematic reviews, no separate analysis was performed, differentiating between internal and external or various internal cooling methods. Therefore, this review aims to systematically screen and evaluate the literature on the effects of *internal* cooling on various outcomes (*n* = 11), including performance, physiological, and perceptional parameters. Several previous reviews assessed the effect of cooling on aerobic performance without the differentiation between performance and capacity (Jones et al., 2012; Bongers et al., 2015; Douzi et al., 2019; Jeffries and Waldron, 2019; Zhang, 2019; Bongers et al., 2020). However, aerobic performance relates to completing a certain task as fast as possible (e.g., time trials), whereas endurance capacity refers to the exercise time to volitional fatigue at a constant workload or speed (e.g., time to exhaustion) (Saris et al., 2003). Endurance capacity is more often studied since the technique is relatively easy to control, and the constant workload allows comparison of metabolic and other measurements between intervention and control trials. Yet, for the assessment of true aerobic performance, time trials are the more valid and realistic approach since there are only a few events where athletes have to exercise as long as possible (Saris et al., 2003). Therefore, in this systematic review, the effect of internal cooling on performance was further differentiated by the exercise protocol used (e.g., time trials vs. time to exhaustion).

## 2 Methods

Data was reported according to the Preferred Reporting Items for Systematic Reviews and Meta-Analyses (PRISMA) statement (Page et al., 2021). The meta-analysis was registered in PROSPERO (no. CRD42022336623).

### 2.1 Search strategy

The following outcomes were considered for the present meta-analysis: (1) performance (time to exhaustion, finish time of time trials, mean power output), (2), physiological (core temperature, skin temperature, sweat rate, heart rate, blood lactate), and (3) perceptional parameters (rate of perceived exertion, thermal sensation, thermal comfort).

A systematic literature search was performed by one researcher (JH) on 17 Dec 2021, using the database of MEDLINE (*via* PubMed). Details of the search strategy can be found in Supplementary Material S1. Keywords included terms related to internal cooling (e.g., ice-slurry, menthol), performance, physiological (e.g., heart rate, sweat rate) and perceptional outcomes (e.g., rate of perceived exertion, thermal sensation), and population (e.g., athletes, active) and were combined by Boolean logic (AND). Articles were limited to human subjects, English or German language, and publication after 1 Jan 2000. In addition, an unsystematic search was performed by screening the full texts of relevant review articles identified through abstract screening of the systematic search for additional references.

### 2.2 Study selection

Studies were selected following a two-step approach. In the first step, two researchers screened the abstracts identified through a database searching for inclusion and exclusion criteria (Supplementary Material S2). The agreement between the two researchers was quantified using kappa statistics (Orwin et al., 1994).

In the second step, full texts of all identified abstracts were retrieved and screened for inclusion and exclusion criteria. Studies were included as long as data for at least one of the above-mentioned outcomes were reported. Only studies with isocaloric or isovolumetric fluid intake in the trials were included to avoid confounding effects on performance. We considered studies conducted in hot environments (>30°C) but also in neutral-warm environments (20°C–30°C). The first author was responsible for the study selection of full texts. A list of excluded articles can be obtained in Supplementary Material S3.

### 2.3 Study classification

After inclusion, the studies were divided according to (1) the time point of cooling relative to exercise [before (pre-), during (mid-), before and during (pre- + mid-)], and (2) cooling method (physical, perceptional). Studies comparing multiple internal cooling interventions with the same control condition were included repeatedly.

For interventions involving the administration of fluid, we further classified the treatment groups according to drink temperature: (1): intervention: beverages with a temperature ≤10°C, (2), control: beverages with a temperature >18 and ≤50°C.

### 2.4 Data extraction, transformations, and quality assessment

The first author extracted all data from the articles’ text or tables and entered them into a synoptical table. Values for the physiological and perceptional outcomes were extracted at exhaustion, at the end of the exercise, or at the last time point reported, except sweat rate, for which we used the total across the whole trial. However, for performance outcomes, we chose the mean value for power output during the performance trial and end-exercise time for time trial or time to exhaustion protocols. Authors of *n* = 45 articles were contacted to receive further data (response rate: 75.6%).

To harmonize data, several transformations were performed (further details in the statistics section): (1): The dose of the used internal cooling method was transformed to mg (menthol) or g · kg^-1^ (ice or cold-water), assuming 1 mL corresponds to 1 g, and by dividing absolute intake by mean body mass. (2) Heat index was calculated using the reported mean ambient temperature and relative humidity by applying the Rothfusz equation developed and adopted by the National Oceanic and Atmospheric Administration (Rothfusz, 1990). Since the equation is invalid for conditions of temperature and relative humidity which warrant a heat index value below about 26.67% (Zune et al., 2020), the reported mean ambient temperature instead of the heat index was chosen for studies reporting ambient temperatures <27°C and relative humidity <40%. In studies reporting wet-bulb globe temperature or no relative humidity, wet-bulb globe temperature or ambient temperature values were entered in the column heat index, respectively. (3) For studies using opposite scales for thermal comfort assessment (lower values indicating more comfortable and higher values more uncomfortable thermal comfort; *n* = 4), the mean value was mirrored onto the mean of the respective scale while the standard deviation remained the same. (4) For the calculation of total exercise duration, the duration of the activity was taken for steady-state exercise [min]. In contrast, the mean of the time needed in the intervention and control groups was calculated for time trials [min].

Two researchers independently assessed the risk of bias according to Cochrane collaboration guidelines (Higgins et al., 2022). The study authors were not contacted to receive further information to confirm the details of their applied methods.

### 2.5 Statistical analysis

Baseline characteristics of each study sample were reported as mean with standard deviation. RevMan 5 (The Cochrane Collaboration, 2020) was used to perform the meta-analysis. Differences between intervention and control with regard to performance, physiological or perceptional parameters were expressed as standardized mean differences (SMD) with 95% Confidence Intervals (CI’s) using the inverse-variance method and a random-effects model. The SMD was chosen because of methodological differences between the studies. Effects were considered as *trivial* (SMD <0.2), *small* (0.2 < SMD <0.5), *medium* (0.5 < SMD <0.8), and *large* (SMD ≥0.8) according to Cohen (Cohen, 1992).

Statistical heterogeneity was assessed by examining forest plots, CI’s, and calculating the I^2^ index. I^2^ values of 25%, 50%, and 75% indicated low, medium, and high heterogeneity, respectively. An I^2^ > 50% demonstrated significant heterogeneity between studies. Funnel plots were used to assess possible bias in reporting and publication (data available upon request from the authors). When likely [i.e., sufficient studies (*n* ≥ 10) (Ryan, 2016)], meta-regression was performed to identify covariates for the dispersion of the main effect size. Possible covariates included dose, heat index, and exercise duration. Meta-regression was performed using SPSS version 23 (IBM Corp., Chicago, IL, United States), and bubble plots of significant regression models were created to visually show associations (Lajeunesse, 2021).

## 3 Results

### 3.1 Study characteristics

**FIGURE 1:**
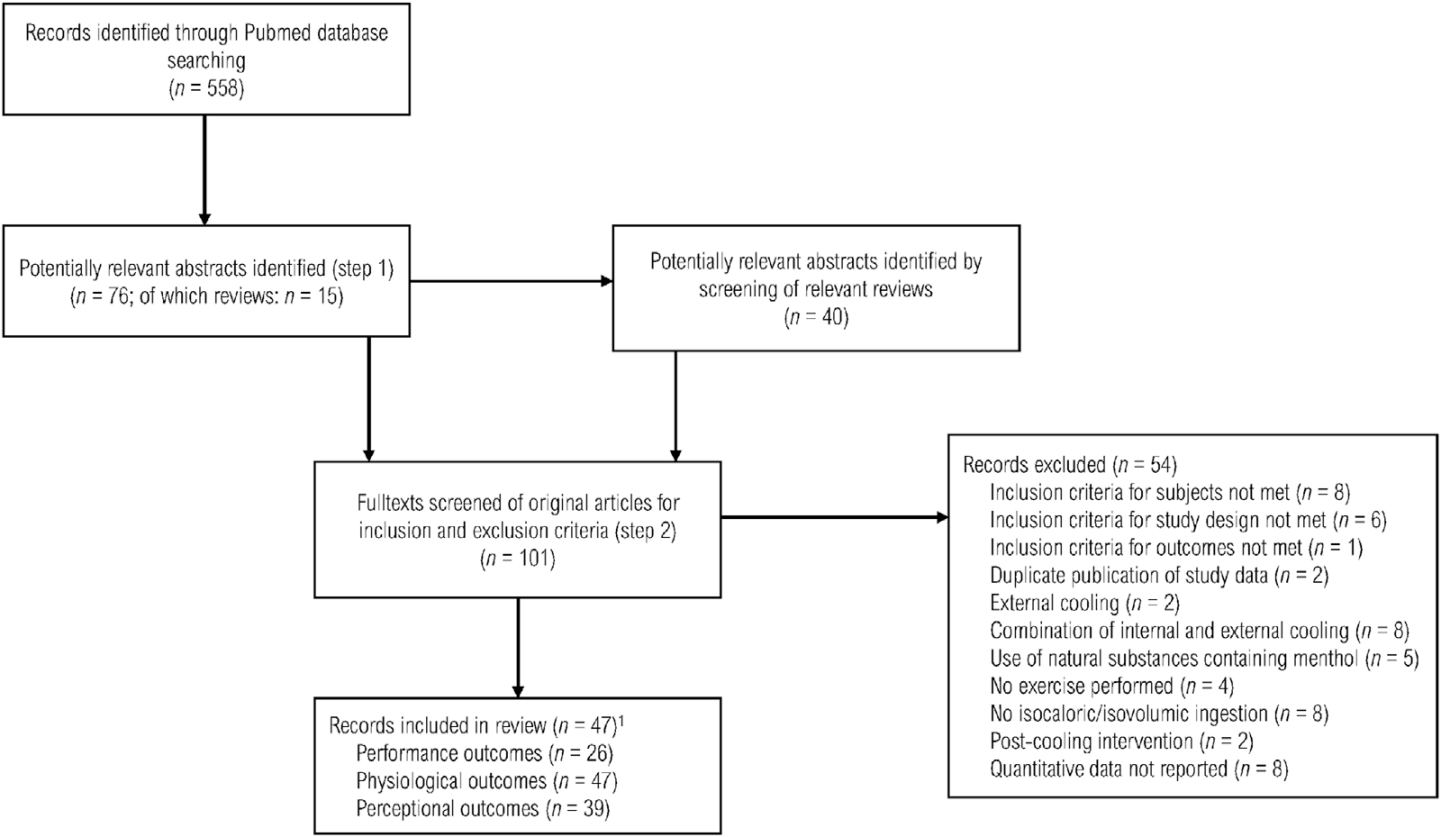
Overview of the selection process of the included studies for this review. *n* indicates the number of studies. ^1^ Total number differs from the sum of subscores as several studies reported multiple outcomes.

Our search identified 558 abstracts. Initially, 101 reports seemed possibly relevant, but after a thorough full-text review, only 47 studies were included (Lee and Shirreffs, 2007; Lee et al., 2008; Lee et al., 2008; Burdon et al., 2010; Ihsan et al., 2010; Stanley et al., 2010; Byrne et al., 2011; Siegel et al., 2011; Bain et al., 2012; Siegel et al., 2012; Burdon et al., 2013; Hue et al., 2013; Brade et al., 2014; Morris et al., 2014; Burdon et al., 2015; Hue et al., 2015; James et al., 2015; Lamarche et al., 2015; Pryor et al., 2015; Schulze et al., 2015; Zimmermann and Landers, 2015; Hailes et al., 2016; Morris et al., 2016; Stevens et al., 2016; Tay et al., 2016; Flood et al., 2017; Gerrett et al., 2017; Takeshima et al., 2017; Zimmermann et al., 2017; Zimmermann et al., 2017; Jeffries et al., 2018; Ng et al., 2018; Snipe and Costa, 2018; Watkins et al., 2018; Aldous et al., 2019; Gibson et al., 2019; Ng et al., 2019; Thomas et al., 2019; Iwata et al., 2020; Naito et al., 2020; Nakamura et al., 2020; Onitsuka et al., 2020; Saldaris et al., 2020; Alhadad et al., 2021; Gavel et al., 2021; Parton et al., 2021; Tabuchi et al., 2021). The kappa value of 0.76 for the agreement between the two researchers assessing the eligibility of records was considered to reflect a “substantial” agreement (Orwin et al., 1994). Figure 1 displays a PRISMA flow chart of the literature search. A description of the included studies is given in Supplementary Material S4.

Data of 486 subjects (13.7% female) with a reported mean age between 20 and 42 years were included. Ambient conditions ranged between 22°C–49.6°C and 15.4%–80% relative humidity.

In total, *n* = 17, *n* = 16, and *n* = 9 studies assessed the effect of pre-, mid-, and pre- + mid-exercise ice/cold-water cooling, respectively. For menthol, *n* = 4 and *n* = 3 studies used pre-, and pre- + mid-exercise cooling, respectively. The dose of ice/cold-water ingestion ranged from 1.25–30 g kg^-1^. All included studies applying menthol used mouth rinsing instead of ingestion. Therefore, in the following, the term menthol mouth rinsing will be used. The accumulated dose of menthol mouth rinsing was in the range of 2.5–200 mg.

The correlation for the SMD with dose, heat index, and exercise duration was only calculated for ice/cold-water ingestion, and correlations were calculated for all outcomes except for time trial performance and blood lactate. For menthol mouth rinsing, the number of studies was insufficient for correlation analyses.

### 3.2 Risk of bias in the included studies and heterogeneity

The studies included generally had, dependent on the category, a low, unclear, or high risk of bias (Supplementary Material S5). Only two studies reported information on the randomization procedure conducted to generate groups (Flood et al., 2017; Jeffries et al., 2018). No study reported attempts to conceal allocation to an intervention or control group; therefore, the risk of bias was considered “high”. Only one study reported double-blinding of participants and personnel to the interventions administered (Parton et al., 2021); two studies were single-blinded (Flood et al., 2017; Jeffries et al., 2018). The remaining studies did not report any blinding; therefore, the risk of bias in the outcome measure was estimated as “high”. However, we acknowledge that blinding of internal cooling is difficult due to the distinctive sensory properties of menthol and ice/cold-water. All studies were assigned a “low” risk of attrition bias, since there were either no missing data or missing data were balanced across the intervention groups. Finally, in all but eight studies (Byrne et al., 2011; Siegel et al., 2011; Hue et al., 2015; Lamarche et al., 2015; Hailes et al., 2016; Gerrett et al., 2017; Watkins et al., 2018; Alhadad et al., 2021), outcome data were reported incompletely in the original article, so not all results were entered into the meta-analysis and these studies were rated with a “high” risk for reporting bias. However, most authors provided additional data upon request. Another reason for the high percentage of studies rated with “high” risk for reporting bias is the large number of outcomes (*n* = 11) considered in this study. For example, several studies reported sufficient data for performance but not for all physiological and perceptional outcomes.

According to I^2^ values, the total and subgroup heterogeneity for all outcomes was indicated as low to medium. In addition, the funnel plots showed no bias in reporting and publication. Therefore, no further sensitivity analyses were performed.

### 3.3 Effectiveness of internal cooling on performance

**FIGURE 2:**
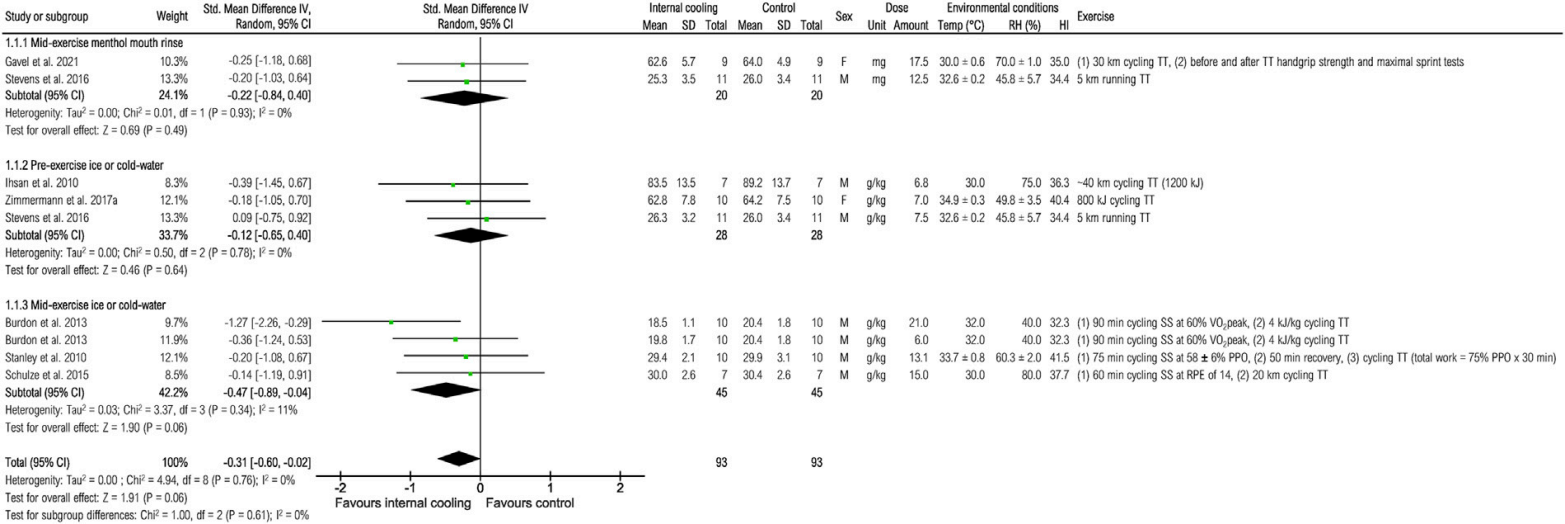
Meta-analysis of standardized mean difference in time trial performance [min] with 95% CI between internal cooling and control. F, female; HI, heat index; M, male; PPO, peak power output; RH, relative humidity; RPE, rate of perceived exertion; SS, steady-state exercise; Temp, ambient temperature; TT, time trial; VO_2_peak, peak oxygen consumption.

Seven studies were included to assess the effects of internal cooling on time trial performance (Figure 2). Internal cooling resulted in a *borderline* significant reduction in time trial performance [SMD (95% CI) −0.31 [−0.60; −0.02), *p* = 0.06]. The effect is mainly explained by the application of mid-exercise ice or cold-water, which resulted in a *borderline* significant reduction in time trial performance [−0.47 (−0.89; −0.04), *p* = 0.06], whereas non-significant effects were obtained for pre-exercise ice or cold-water and menthol mouth rinsing (all *p* > 0.05).

**FIGURE 3:**
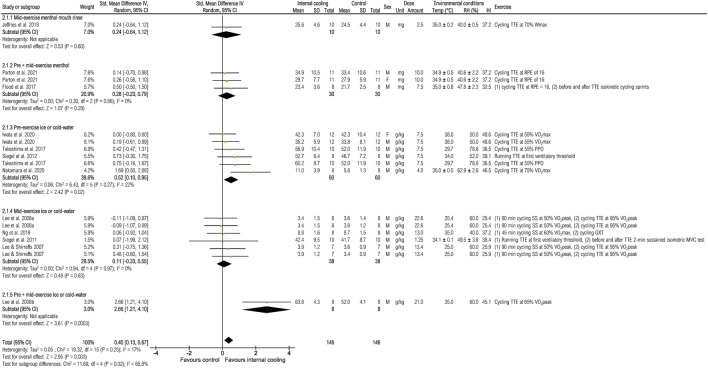
Meta-analysis of standardized mean difference in time to exhaustion [min] with 95% CI between internal cooling and control. F, female; GXT, graded exercise test; HI, heat index; M, male; MVC, maximum voluntary contraction; PPO, peak power output; RH, relative humidity; RPE, rate of perceived exertion; SS, steady-state exercise; Temp, ambient temperature; TTE, time to exhaustion; VO_2_max, maximum oxygen consumption; VO_2_peak, peak oxygen consumption; Wmax, maximum power.

Twelve studies were included to assess the effects of internal cooling on time to exhaustion (Figure 3). There was a significant positive *small* effect of internal cooling on time to exhaustion when pooling all studies [0.40 (0.13; 0.67), *p* < 0.01]. However, the subgroup analysis showed that only pre-exercise application of ice or cold-water resulted in a significant positive *moderate* effect [0.52 (0.10; 0.95), *p* < 0.05].

**FIGURE 4:**
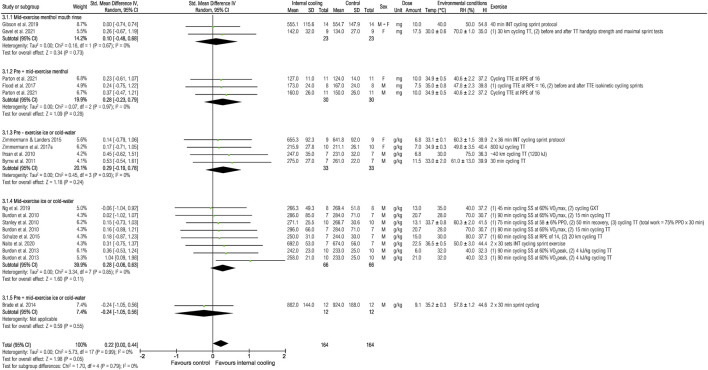
Meta-analysis of standardized mean difference in mean power output [W] with 95% CI between internal cooling and control. F, female; GXT, graded exercise test; HI, heat index; INT, intermittent exercise; M, male; PPO, peak power output; RH, relative humidity; RPE, rate of perceived exertion; SS, steady-state exercise; Temp, ambient temperature; TT, time trial; TTE, time to exhaustion; VO_2_max, maximum oxygen consumption; VO_2_peak, peak oxygen consumption.

Fifteen studies were included to assess the effects of internal cooling on mean power output (Figure 4). When pooling all studies, a *borderline* significant positive effect of internal cooling on mean power output was observed [0.22 (0.00; 0.44), *p* = 0.05]. When looking at subgroup analysis, there were no significant effects for specific internal cooling methods (all *p* > 0.05).

### 3.4 Effectiveness of internal cooling on physiological parameters

**FIGURE 5:**
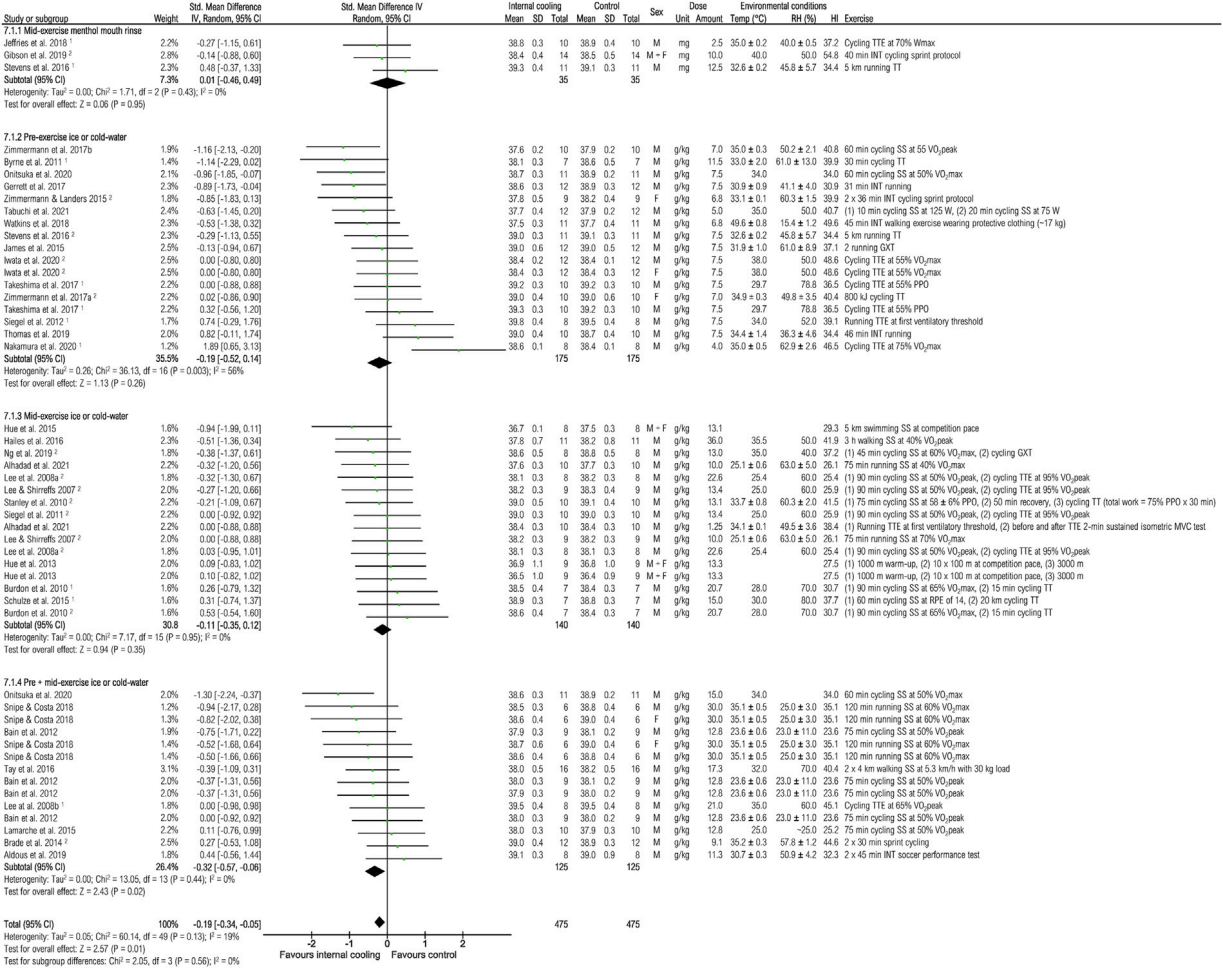
Meta-analysis of standardized mean difference in core temperature [°C] with 95% CI between internal cooling and control. ^1^ Studies with significant positive performance effects of internal cooling. ^2^ Studies with no performance effects of internal cooling. F, female; GXT, graded exercise test; HI, heat index; INT, intermittent exercise; M, male; MVC, maximum voluntary contraction; PPO, peak power output; RH, relative humidity; RPE, rate of perceived exertion; SS, steady-state exercise; Temp, ambient temperature; TT, time trial; TTE, time to exhaustion; VO_2_max, maximum oxygen consumption; Wmax, maximum power.

Thirty-five studies were included to assess the effects of internal cooling on core temperature at the end of exercise (Figure 5). Internal cooling resulted in a significant reduction in core temperature, with the effect considered *trivial* [−0.19 (−0.34; −0.05), *p* < 0.05]. However, the subgroup analysis showed that only pre-plus mid-exercise application of ice or cold-water resulted in a significant reduction of core temperature with *small* effect [−0.32 (−0.57; −0.06), *p* < 0.05].

**FIGURE 6:**
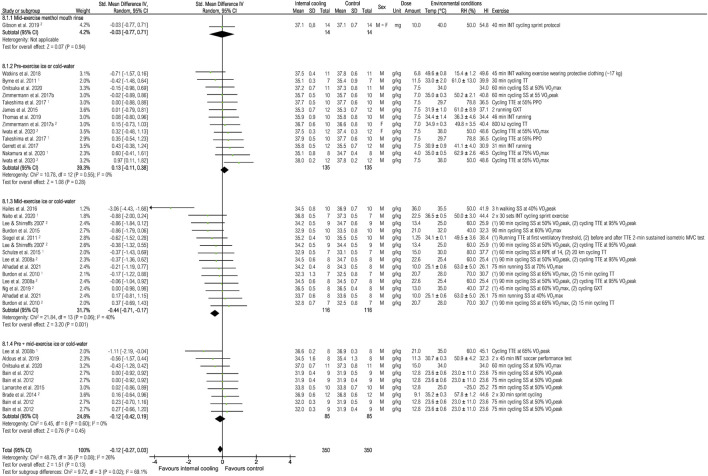
Meta-analysis of standardized mean difference in skin temperature [°C] with 95% CI between internal cooling and control. ^1^ Studies with significant positive performance effects of internal cooling. ^2^ Studies with no performance effects of internal cooling. F, female; GXT, graded exercise test; HI, heat index; INT, intermittent exercise; M, male; MVC, maximum voluntary contraction; PPO, peak power output; RH, relative humidity; RPE, rate of perceived exertion; SS, steady-state exercise; Temp, ambient temperature; TT, time trial; TTE, time to exhaustion; VO_2_max, maximum oxygen consumption; VO_2_peak = peak oxygen consumption.

Twenty-seven studies were included to assess the effects of internal cooling on skin temperature at the end of exercise (Figure 6). No effect of internal cooling on skin temperature were observed when pooling all studies (−0.12 [-0.27; 0.03], *p* = 0.13). However, mid-exercise application of ice or cold-water resulted in a significant reduction of skin temperature with *small* effect [−0.44 (−0.71; −0.17), *p* < 0.01].

**FIGURE 7:**
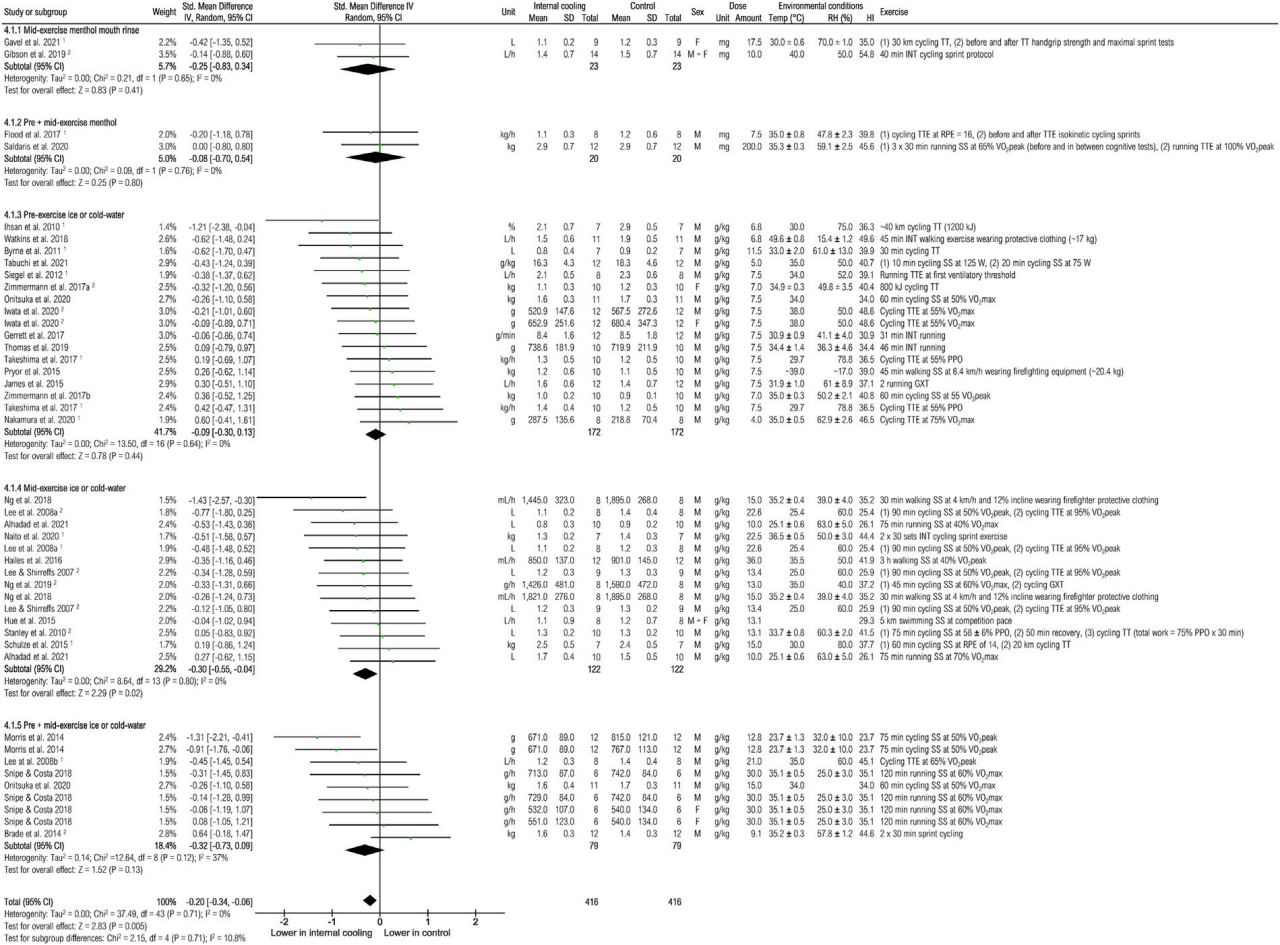
Meta-analysis of standardized mean difference in sweat rate with 95% CI between internal cooling and control. ^1^ Studies with significant positive performance effects of internal cooling. ^2^ Studies with no performance effects of internal cooling. F, female; GXT, graded exercise test; HI, heat index; INT, intermittent exercise; M, male; MVC, maximum voluntary contraction; PPO, peak power output; RH, relative humidity; RPE, rate of perceived exertion; SS, steady-state exercise; Temp, ambient temperature; TT, time trial; TTE, time to exhaustion; VO_2_max, maximum oxygen consumption; VO_2_peak = peak oxygen consumption.

Thirty-three studies were included to assess the effects of internal cooling on total sweat rate (Figure 7). Internal cooling resulted in a significant reduction in total sweat rate, with the effect considered *small* [−0.20 (−0.34; −0.06), *p* < 0.01]. However, the subgroup analysis showed that only mid- [−0.30 (−0.55; −0.04), *p* < 0.05] application of ice or cold-water resulted in a significant reduction of total sweat rate with *small* effect.

**FIGURE 8:**
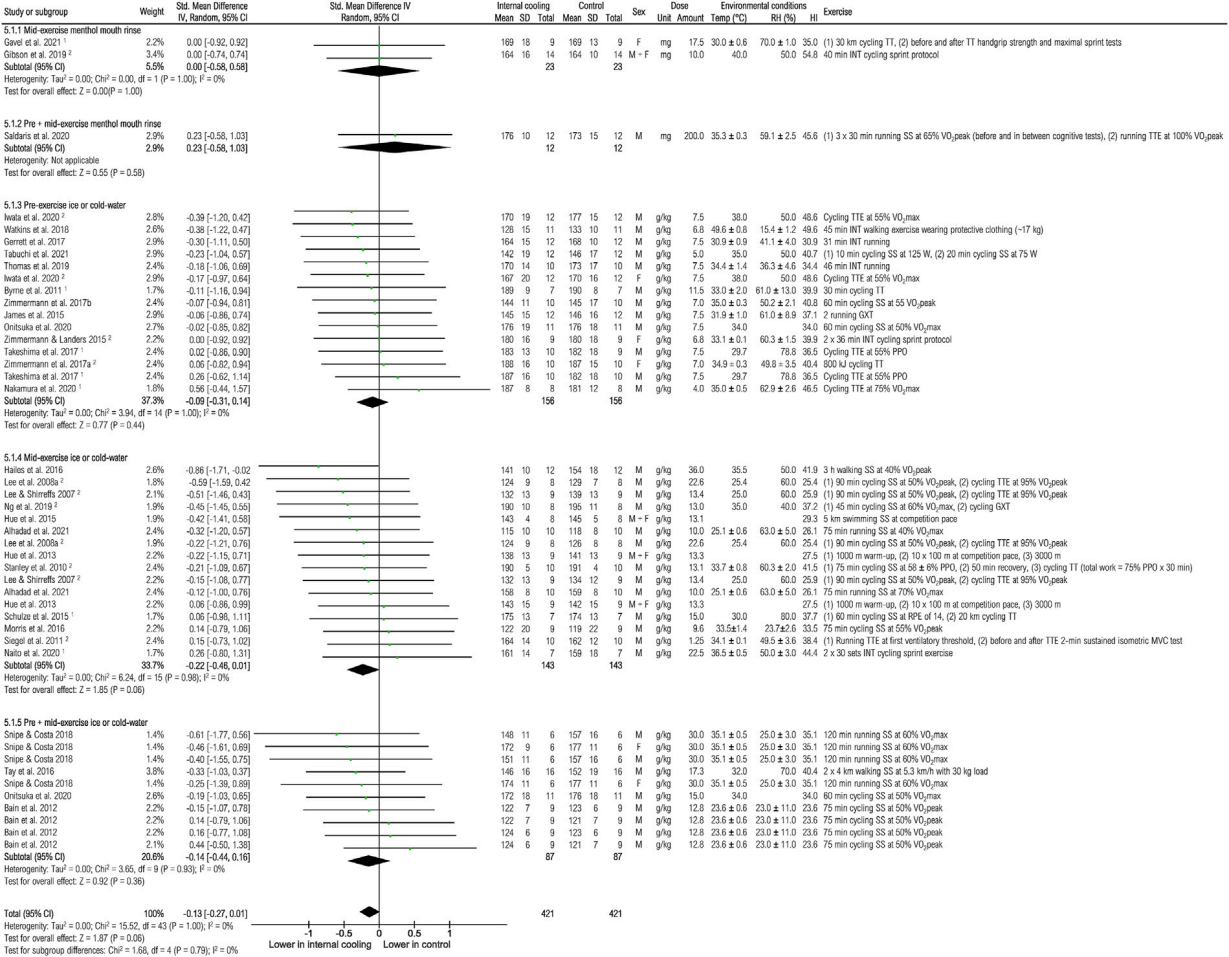
Meta-analysis of standardized mean difference in heart rate [bpm] with 95% CI between internal cooling and control. ^1^ Studies with significant positive performance effects of internal cooling. ^2^ Studies with no performance effects of internal cooling. F, female; GXT, graded exercise test; HI, heat index; INT, intermittent exercise; M, male; MVC, maximum voluntary contraction; PPO, peak power output; RH, relative humidity; RPE, rate of perceived exertion; SS, steady-state exercise; Temp, ambient temperature; TT, time trial; TTE, time to exhaustion; VO_2_max, maximum oxygen consumption; VO_2_peak = peak oxygen consumption.

Thirty-one studies were included to assess the effects of internal cooling on heart rate at the end of exercise (Figure 8). Internal cooling resulted in a *borderline* significant reduction in heart rate [−0.13 (−0.27; 0.01), *p* = 0.06]. Subgroup analysis revealed that mid-exercise application of ice or cold-water resulted in a *borderline* reduction of heart rate [−0.22 (−0.46; 0.01), *p* = 0.06].

**FIGURE 9:**
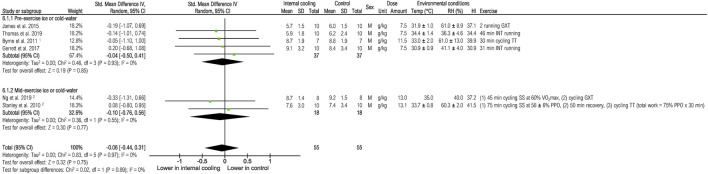
Meta-analysis of standardized mean difference in blood lactate [mmol/L] with 95% CI between internal cooling and control. ^1^ Studies with significant positive performance effects of internal cooling. ^2^ Studies with no performance effects of internal cooling. F, female; GXT, graded exercise test; HI, heat index; INT, intermittent exercise; M, male; PPO, peak power output; RH, relative humidity, SS, steady-state exercise; Temp, ambient temperature; TT, time trial; VO_2_max, maximum oxygen consumption.

Six studies were included to assess the effects of internal cooling on blood lactate at the end of exercise (Figure 9). No effects of internal cooling on blood lactate were observed when pooling all studies [−0.06 (−0.44; 0.31), *p* = 0.75] or performing subgroup analysis (all *p* > 0.05).

### 3.5 Effectiveness of internal cooling on perception

**FIGURE 10:**
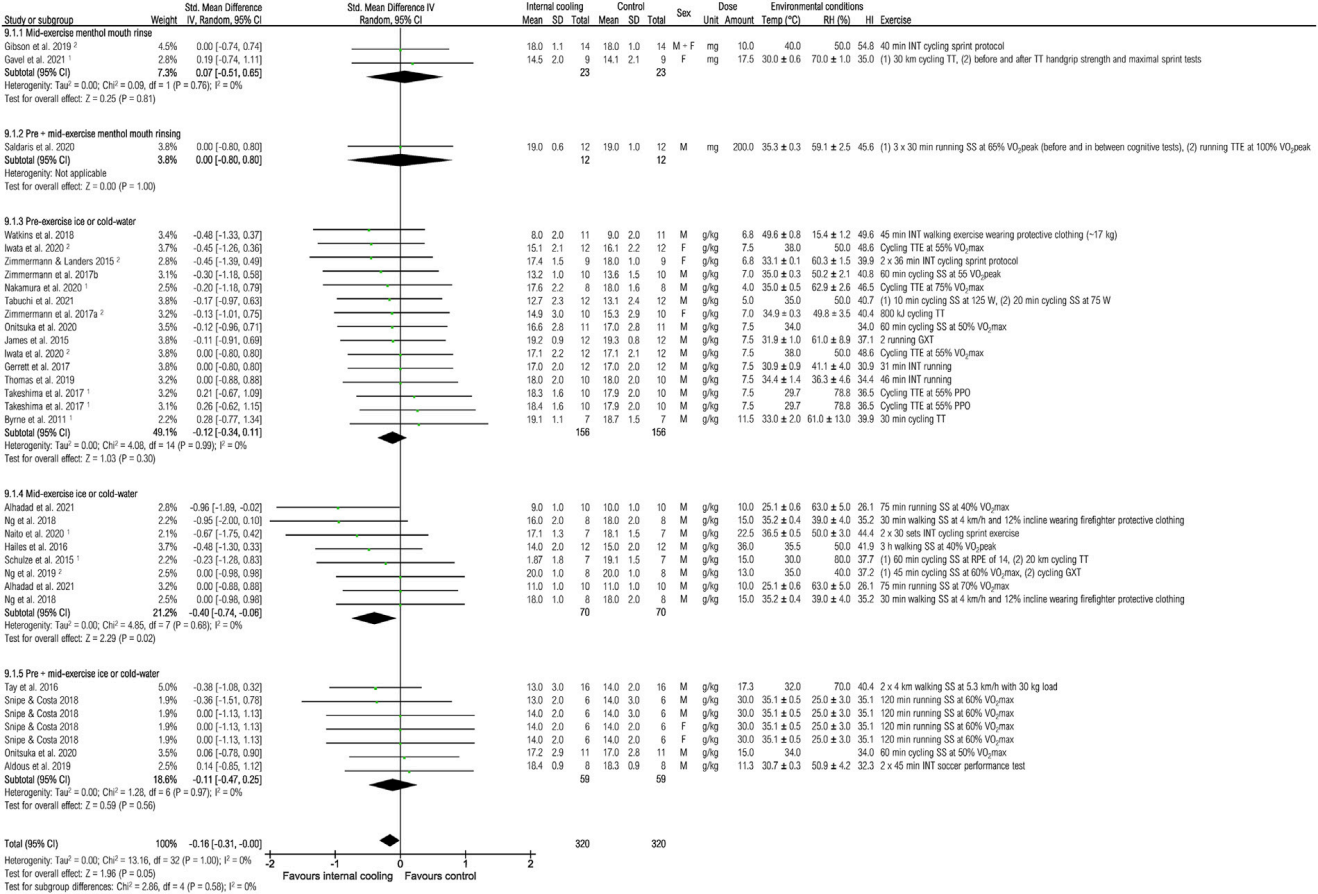
Meta-analysis of standardized mean difference in rate of perceived exertion with 95% CI between internal cooling and control. ^1^ Studies with significant positive performance effects of internal cooling. ^2^ Studies with no performance effects of internal cooling. F, female; GXT, graded exercise test; HI, heat index; INT, intermittent exercise; M, male; PPO, peak power output; RH, relative humidity; RPE, rate of perceived exertion; SS, steady-state exercise; Temp, ambient temperature; TT, time trial; TTE, time to exhaustion; VO_2_max, maximum oxygen consumption; VO_2_peak, peak oxygen consumption.

Twenty-five studies were included to assess the effects of internal cooling on rate of perceived exertion at the end of exercise (Figure 10). Internal cooling resulted in a *borderline* significant reduction of rate of perceived exertion [−0.16 (−0.31; −0.00), *p* = 0.05]. However, the subgroup analysis showed that mid-exercise application of ice or cold-water resulted in a significant reduction of rate of perceived exertion with *small* effect [−0.40 (−0.74; −0.06), *p* < 0.05].

**FIGURE 11:**
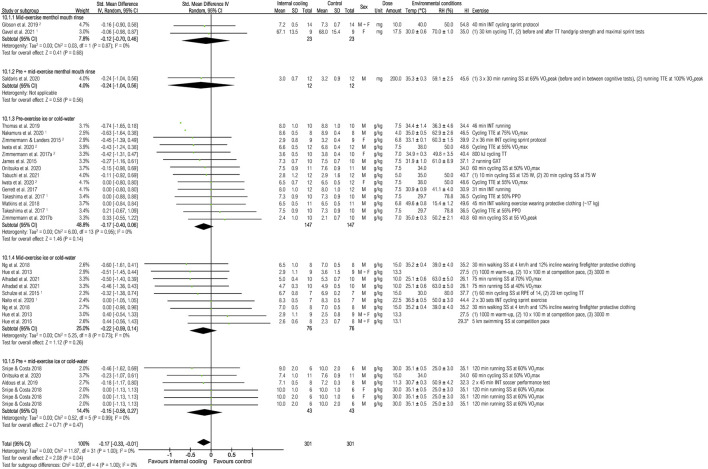
Meta-analysis of standardized mean difference in thermal sensation with 95% CI between internal cooling and control. ^1^ Studies with significant positive performance effects of internal cooling. ^2^ Studies with no performance effects of internal cooling. F, female; GXT, graded exercise test; HI, heat index; INT, intermittent exercise; M, male; PPO, peak power output; RH, relative humidity; RPE, rate of perceived exertion; SS, steady-state exercise; Temp, ambient temperature; TT, time trial; TTE, time to exhaustion; VO_2_max, maximum oxygen consumption; VO_2_peak, peak oxygen consumption.

Twenty-three studies were included to assess the effects of internal cooling on thermal sensation at the end of exercise (Figure 11). Internal cooling resulted in a significant reduction of thermal sensation, with the effect considered *trivial* {−0.17 [−0.40 (−0.74; −0.06), *p* < 0.050.33; −0.01], *p* < 0.05}. However, no internal cooling method resulted in significant effects when looking at subgroup analysis (all *p* > 0.05).

**FIGURE 12:**
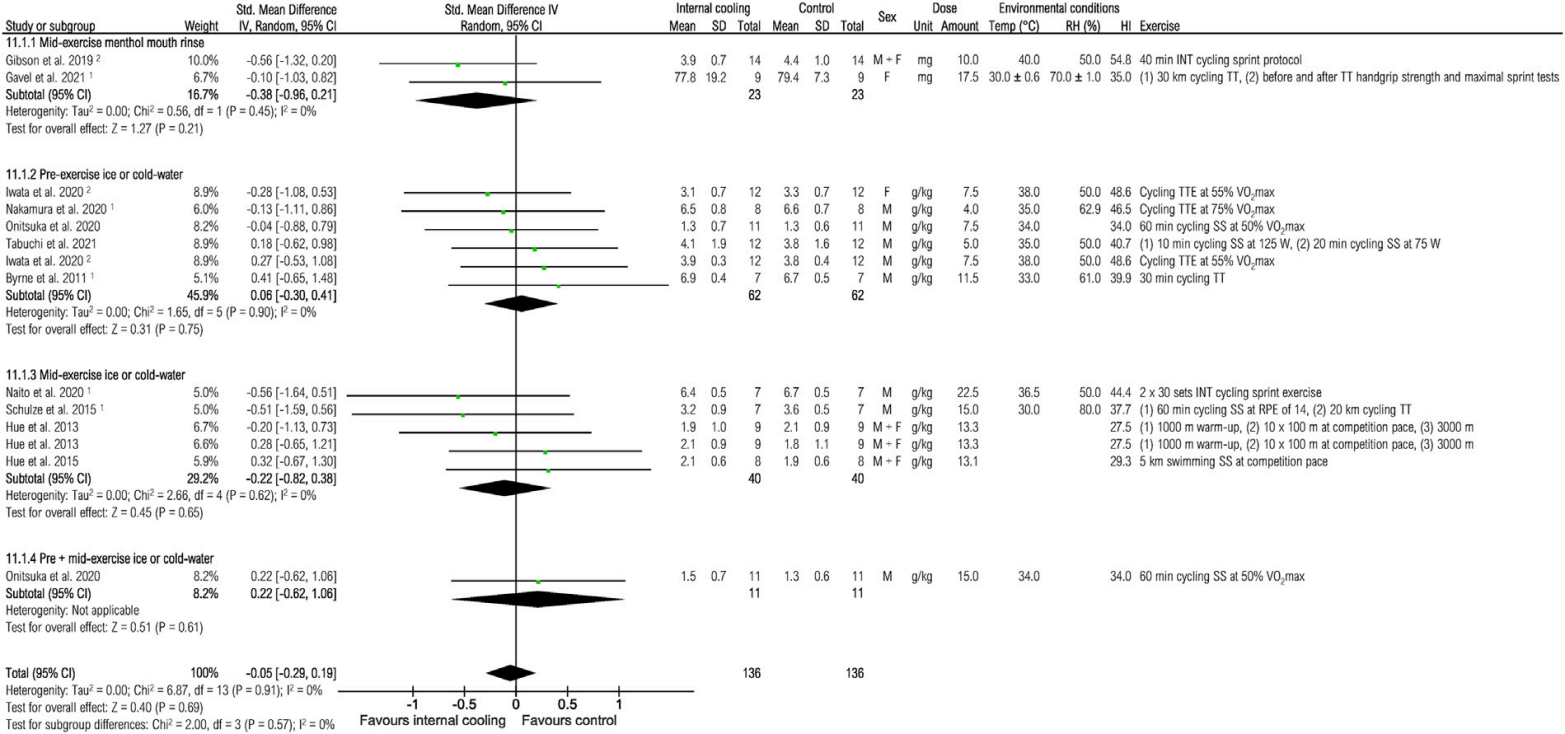
Meta-analysis of standardized mean difference in thermal comfort with 95% CI between internal cooling and control. ^1^ Studies with significant positive performance effects of internal cooling. ^2^ Studies with no performance effects of internal cooling. F, female; HI, heat index; INT, intermittent exercise; M, male; RH, relative humidity; RPE, rate of perceived exertion; SS, steady-state exercise; Temp, ambient temperature; TT, time trial; TTE, time to exhaustion; VO_2_max, maximum oxygen consumption.

Eleven studies were included to assess the effects of internal cooling on thermal comfort at the end of exercise (Figure 12). No effects of internal cooling on thermal comfort were observed when pooling all studies [−0.05 (−0.29; 0.19), *p* = 0.69] or performing subgroup analysis (all *p* > 0.05).

### 3.6 Relationship between SMD and dose, heat index, and exercise duration

**FIGURE 13:**
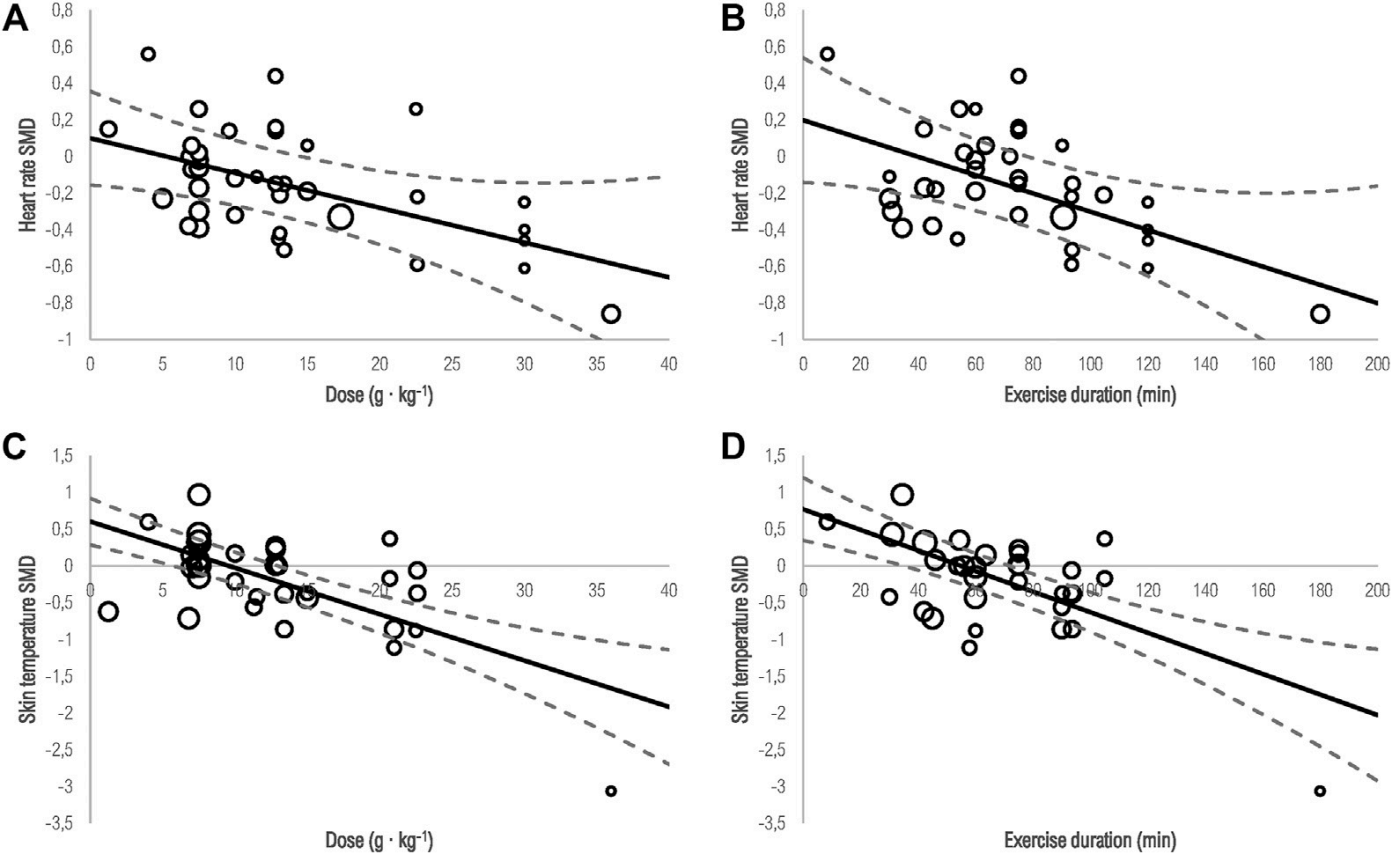
Meta-regression analyses exploring potential heterogenity of heart rate as a result of **(A)** dose, **(B)** exercise duration; and skin temperature as a result of **(C)** dose, **(D)** exercise duration. The bubbles are drawn with sizes proportional to the weight of individual studies. The solid line represents linear predicitions for the effect size while the curved lines represent lower and upper 95% CIs.

Meta-regressions were performed only for ice/cold-water internal cooling, as data for menthol cooling was insufficient for regression analyses. Furthermore, due to the limited number of studies, we did not differentiate between time points of administration (i.e., pre-vs. mid-vs. pre- + mid-exercise). The results of meta-regressions between the SMD of ice/cold-water internal cooling for performance, physiological, and perceptional outcomes, dose, heat index, and exercise duration are shown in Supplementary Material S6. In Figure 13, significant associations are shown as bubble plots. Heart rate and skin temperature SMD were significantly associated with the dose and exercise duration (all *p* < 0.01). There were no significant associations between heat index and SMDs for heart rate (*p* = 0.96) and skin temperature (*p* = 0.55). There was a *borderline* significant association between time trial performance SMD and dose (*p* = 0.09) and for sweat rate SMD and heat index (*p* = 0.08). No significant associations with dose, heat index, and exercise duration were observed for all other outcomes (*p* > 0.05).

## 4 Discussion

The purpose of the present review and meta-analysis was to systematically analyze and quantify the effects of internal cooling methods on performance and physiological as well as perceptional parameters while exercising in the heat. Our main findings demonstrate that internal cooling improves physical performance and reduces overall sweat rate, core temperature and thermal sensation at the end of the exercise. These effects, however, depend on the method (physical vs. perceptional cooling) as well as the time of administration (pre-vs. mid-vs. pre- and mid-cooling).

Our main finding of the present study was that internal cooling resulted in improvements in physical performance, which is in agreement with some (Bongers et al., 2015; Zhang, 2019; Rodríguez et al., 2020) but not all (Jones et al., 2012; Ruddock et al., 2017; Choo et al., 2018) systematic reviews on the topic. We overcame this apparent discrepancy in the literature by differentiating between time to exhaustion, typically used as a measure of aerobic capacity, and time trial performance, considered a more realistic and valid measure of performance (Saris et al., 2003). Along this line, we found that time to exhaustion was significantly prolonged by internal cooling, whereas the effect of internal cooling on time trial performance was only *borderline* significant. The positive impact on aerobic capacity is likely linked to our finding of improved perceptional outcomes, such as a lower rate of perceived exertion. For example, in several studies in which cold water or ice ingestion resulted in a significantly longer time to exhaustion (Lee et al., 2008b; Siegel et al., 2012), the authors reported lower rates of perceived exertion during exercise, but notably not at the end of the exercise bout. Considering that subjective exertion is closely linked to the time to exhaustion (Presland et al., 2005), lower rates of perceived exertion, as seen in our analysis, likely allowed the subjects to exercise for a longer duration until exhaustion was achieved. Thermal sensation, which is an essential mediator of behavioral thermoregulation that integrates with the rate of perceived exertion as the predominant controller of the self-selected work rate of exercise (Flouris and Schlader, 2015), was also significantly reduced in our analysis, suggesting that performance improvements are likely linked to lower sensations of heat and exertion.

In addition to changes in exertion perception, physiological outcomes likely explain performance improvements. Our meta-analysis demonstrated significant reductions in core temperature and sweat rate and a *borderline* significant reduction in heart rate following internal cooling. Ingesting (ice-) cold beverages will lower core temperature as considerable amounts of internal heat will be absorbed, and a consequent delay in the onset of thermally induced fatigue might occur (Wegmann et al., 2012). In addition, brain temperature might be reduced (Onitsuka et al., 2018), increasing the probability of thermal sensation and performance improvements during later stages of exercise. Due to the activation of gastrointestinal thermoreceptors (Villanova et al., 1997), positive effects on the inhibitory feedback on core temperature and subsequent performance improvements might occur. However, several studies reported no differences or even reductions (Byrne et al., 2011) in core temperature, although exercise intensity was higher or exercise duration prolonged (Lee et al., 2008b; Burdon et al., 2010; Schulze et al., 2015; Takeshima et al., 2017) after internal cooling. This finding might be explained by the fact that athletes were able to perform at higher exercise intensity when applying internal cooling. Therefore, internal heat production might be greater, affecting physiological and perceptional outcomes. Since we chose to limit our analysis of physiological and perceptional outcomes to measurements taken at the *end* of the exercise, our results might be diluted by differences in exercise intensities or duration. We can conclude that studies showing no differences in end-exercise physiological outcomes with higher exercise intensity or duration support the positive effects of the internal cooling intervention on physiological parameters. In contrast, findings of increased core temperature at exhaustion following internal physical cooling are likely due to higher intensity or longer exercise duration in the trial (Siegel et al., 2012; Nakamura et al., 2020). When interpreting the impact of internal cooling on physiological or perceptional outcomes, it is therefore always crucial to take into account the time course and their relationship with exercise intensity and performance.

In contrast to previous systematic reviews (Ruddock et al., 2017; Choo et al., 2018), we observed a significant reduction in whole-body sweat rate following internal cooling. According to Morris et al. (Morris et al., 2016), human abdominal thermoreceptors detect intra-abdominal temperature changes, and due to their sufficient integration within the central nervous system, they can further elicit thermoeffector responses at the skin surface. A reduction of whole-body sweat rate and further evaporative heat loss from the skin might result in a lower, rather than greater, net heat loss and subsequently a greater heat storage during exercise (Morris et al., 2016). Therefore, the authors recommend to ingest beverages of any temperature, but not ice-cold drinks, during competition in hot and *dry* environments, where evaporative heat loss plays a greater role in total heat dissipation (Morris et al., 2016). On the other side, a lower core temperature likely reduces the sweat rate necessary for cooling (Montain et al., 1995). A lower sweat rate might further reduce the risk of dehydration. Since performance impairments might occur with sweat loss rates >2–4% of body mass (Thomas et al., 2016), the performance improvements of internal cooling might also be partially explained by lower sweat loss.

In the present study, we found only a *borderline* significant reduction in heart rate following internal cooling. These results concur with previous systematic reviews, which reported no internal cooling effect on heart rate (Bongers et al., 2015; Ruddock et al., 2017; Choo et al., 2018). As for other physiological and perceptional outcomes, this discrepancy might be explained by our inclusion of data collected at the end of the exercise only. Further, our results do not imply there were no positive effects of internal cooling on skin blood flow or stroke volume, as heart rate in this context is only an index of these variables (Ruddock et al., 2017). Since, in the present study, we did not include skin blood flow, an essential factor for thermoregulation, as an outcome, our analysis does not provide conclusive evidence about the underlying physiological mechanisms related to reductions in sweat and heart rate.

Physical cooling seems more effective than perceptional cooling in improving physiological parameters and physical performance when comparing cooling strategies. In agreement with another meta-analysis (Keringer et al., 2020), we found no effects of perceptional cooling on physiological outcomes, as menthol is a non-thermal cooling stimulus that acts on thermoreceptors, inducing sensations of coolness without physical reductions in body temperature (Watson et al., 1978). We further found no effects of menthol cooling on performance, which is in line with two previous (Douzi et al., 2019; Keringer et al., 2020) but in contrast with one meta-analysis (Jeffries and Waldron, 2019). The positive performance effects of perceptional cooling reported in some studies may probably be due to previously reported changes in perceptional outcomes (Jeffries and Waldron, 2019; Keringer et al., 2020), which we were also unable to demonstrate in our analysis. These perceptional effects are likely caused by an activation of cold sensors, leading to reduced thermal sensation and physiological reactions similar to physical cooling (Zheng, 2013).

The time point of application might be an essential factor in evaluating the efficacy of internal cooling. We found that cooling before *and* during exercise significantly reduces core temperature, which can be explained by the continuous facilitation of heat storage capacity and extended exercise duration in the heat (Siegel and Laursen, 2012). Our finding that pre- or mid-physical cooling did not reduce core temperature might be explained by the aforementioned limitation to outcome data recorded only at the end of the exercise. However, it is also possible that the effects of pre-cooling might already disappear throughout the exercise. The time point of cooling also impacted effects on perceived exertion, which was reduced only for mid-exercise cooling. Further, physical cooling *during* exercise seems more effective in improving aerobic performance, whereas ingestion *before* exercise may be more beneficial to increase aerobic capacity. Taken together, the most benefits are likely to occur when cooling before *and* during exercise.

Although the benefits of internal cooling on physical performance have been demonstrated in several studies and were confirmed in the present analysis, the optimal dose and time point of ingestion remain unclear. Usually, a total dose of ∼500–700 mL of ice/cold-water (∼7.5 g · kg-1), divided into smaller amounts (∼1.25 g · kg-1 every 5 min until reaching the total dose), is recommended to offer greater cooling and better tolerance (Naito et al., 2017). In the present analysis, total doses ranged from 1.25–30 g kg^-1^, and positive performance effects have been reported even in studies at the lower end of the spectrum (4.0–6.8 g kg^-1^) (Ihsan et al., 2010; Burdon et al., 2013; Nakamura et al., 2020). Our meta-regression failed to confirm the previously reported dose-response effect of physical cooling on performance (Zhang, 2019), as the relationship between dose and improved time trial performance SMD was only *borderline* significant. Regardless, the study with the largest dose (∼21 g · kg-1) had the greatest positive effect on time trial performance (Burdon et al., 2013), also indicating that even with higher doses, the positive effect of cooling may outmatch possibly negative effects of weight gain due to increased fluid intake. However, athletes should always consider that overdrinking increases the risk for hyponatremia, causing several health and performance impairments (Thomas et al., 2016). Ingestion of cold drinks might also increase voluntary fluid consumption during exercise in the heat (Mündel et al., 2006). A greater volume of cold fluid might further act as a heat sink, thereby reducing heat stress’s effects and possibly increasing the time needed to reach an exercise-limiting core temperature. Furthermore, higher voluntary fluid ingestion might reduce the risk of dehydration and might have a beneficial impact on physical performance (Thomas et al., 2016). In conclusion, further studies are needed to determine the dose-response relationship with performance and what the minimum and maximum doses for performance improvements are.

Our results support previous findings (Zhang, 2019) that physical cooling improves performance independent of environmental conditions. These results indicate that internal cooling might already be effective in neutral-warm environments (20°C–30°C). Furthermore, our results show that physical cooling might improve aerobic performance independent of the exercise duration. Our results imply that even athletes exercising with high intensity and short duration might benefit from internal physical cooling.

Considering that most studies showed no beneficial effect of internal perceptional cooling on core and skin temperature while exercise intensity is increased, one might speculate that perceptional cooling could increase the risk of heat-related illness and non-desirable cardiovascular events (Gillis et al., 2010; Barwood et al., 2020). However, other authors claim that internal perceptional cooling with menthol is an effective and safe method to improve performance without adverse effects (Keringer et al., 2020). Menthol has toxic properties, and an acceptable daily intake value of 0–4 mg kg^-1^ body mass was allocated (World Health Organization, 2019). According to the FAO/WHO Expert Committee on Food Additives the highest estimated dietary exposure of menthol is ∼51 mg d^-1^, estimated by the maximized survey-derived intake (MSDI) method (World Health Organization, 2019). When menthol is used as a flavoring agent (as in mouth rinsing) at current intake levels, no safety concerns are raised (World Health Organization, 2019; Barwood et al., 2020). Therefore, the safety of internal menthol application, including dosage, likely depends on whether the substance enters the human body or not. Athletes wishing to use menthol cooling should be familiar with safe intake protocols, which should be well-practiced prior to competitive use (Barwood et al., 2020). However, the lack of beneficial effects on performance and the risk of adverse side effects (low, but possible) imply that menthol mouth rinsing should not be applied until further evidence is available.

### 4.1 Strengths and limitations

To our knowledge, this is the first systematic review integrating the effects of various internal cooling applications on performance as well as physiological and perceptional outcomes while exercising in the heat. However, we acknowledge several limitations. For example, to maximize the standardization of our data, we limited most outcomes to the last time point of reported data (i.e., at the end of the exercise). And while exercise intensity might be highest at this time point, we may have omitted effects during earlier stages of exercise. However, we considered end-exercise outcomes integrating data across the intervention period as most relevant for athletic performance. Unfortunately, not all studies reported data suitable for inclusion in our meta-analysis. Although we contacted the authors to receive further data for the meta-analysis and had a relatively high response, numerous data were unavailable, and the reporting bias in most studies was considered high.

Furthermore, most studies did not blind their interventions, leading to an increased risk for performance bias, although we acknowledge that conducting double-blind experiments involving cooling is challenging, if not impossible. Most of the included studies were performed in a laboratory setting, with male endurance-trained subjects and no adequate placebo condition. Future studies with adequate experimental design and blinding are required to assess the effects of menthol cooling in field-based sporting contexts, female and elite athletes, and sports other than endurance activities.

Finally, although the number of studies investigating perceptional cooling was small, it was well above what is considered the minimum number for meta-analysis, according to the Cochrane Consumers and Communication Review Group (Ryan, 2016).

## 5 Conclusion

Our research highlights that internal cooling has the potential to improve endurance performance and selected physiological and perceptional parameters. However, its effectiveness depends on the method used (i.e., physical vs. perceptional cooling) as well as the time of administration. Our results suggest that physical cooling is more effective for performance improvements than perceptional cooling, although the number of studies assessing the effects of perceptional cooling was low. Further studies are needed to formulate safe intake recommendations and evaluate possible side effects of internal cooling. In addition, more studies are required to assess the impact of internal cooling on exercise performance rather than exercise capacity. Future research should confirm the laboratory-based results in the field setting and involve a more inclusive study demographic with regard to sex and exercise type.

## Data Availability

Publicly available datasets were analyzed in this study. This data can be found here: https://osf.io/7a3mt/files/osfstorage/6429e4c34ecbc41dbe2679bd.
