# Supplementary material for: Effects of internal cooling on physical performance, physiological and perceptional parameters when exercising in the heat: A systematic review with meta-analyses
Source: Front Physiol. 2023 Apr 11;14:1125969. doi: 10.3389/fphys.2023.1125969 (PMC10126464; doi:10.3389/fphys.2023.1125969)
Supplement: Supplementary file 1 [file DataSheet2.PDF]

Supplementary Material 2

Effects of internal cooling on physical performance, physiological and perceptional parameters when exercising in the heat: a systematic review with meta-analyses

Juliane Heydenreich*, Karsten Koehler, Hans Braun, Mareike Grosshauser, Helmut Heseker, Daniel Koenig, Alfonso Lampen, Stephanie Mosler, Andreas Niess, Alexandra Schek, Anja Carlsohn

*** Correspondence:**
Dr. Juliane Heydenreich
juliane.heydenreich@uni-mainz.de

2 Supplementary Data: Inclusion and exclusion criteria for literature search.

**Inclusion criteria**	**Exclusion criteria**
First step (abstract screening)	
- **Study type**: original studies, review articles (including systematic reviews and meta-analyses), position stands<br>- **Subjects**:<br>- healthy and active<br>- age: 18 – 50 years<br>- **Intervention**: application of internal cooling before and/or during exercise<br>- **Date of publication**: published after 2000	- **Subjects**: paralympic athletes, sedentary subjects<br>- **Intervention**:<br>- application of internal cooling after exercise ("post-cooling")<br>- combination of internal and external cooling
Second step (screening of full texts)	
- **Study type**: intervention studies<br>- **Intervention/control trial**:<br>- isocaloric and isovolumetric intake of drinks between trials<br>- drink temperature: ice/cold-water ≤10°C, control condition 18 - ≤50°C<br>- exercise performed in warm (20 – 30 °C) to hot (>30°C) environment<br>- **Outcomes:** at least one of the following outcomes reported (for meta-analysis: mean, SD, number of subjects):<br>- performance (time trial, time to exhaustion, mean power output)<br>- physiological (sweat rate, heart rate, blood lactate, core temperature[1], skin temperature)	- **Intervention**: natural substances containing menthol (e.g., peppermint oil, mint extract)<br>- **Publication**: duplicate data publication

- perceptional (rate of perceived exertion, thermal sensation, thermal comfort)	

Note. [1]Gastrointestinal, core, and rectal temperatures were defined as core temperature
