# Supplementary material for: Effects of internal cooling on physical performance, physiological and perceptional parameters when exercising in the heat: A systematic review with meta-analyses
Source: Front Physiol. 2023 Apr 11;14:1125969. doi: 10.3389/fphys.2023.1125969 (PMC10126464; doi:10.3389/fphys.2023.1125969)
Supplement: Supplementary file 2 [file DataSheet4.pdf]

Supplementary Material 4

Effects of internal cooling on physical performance, physiological and perceptional parameters when exercising in the heat: a systematic review with meta-analyses

Juliane Heydenreich*, Karsten Koehler, Hans Braun, Mareike Grosshauser, Helmut Heseker, Daniel Koenig, Alfonso Lampen, Stephanie Mosler, Andreas Niess, Alexandra Schek, Anja Carlsohn

*** Correspondence:**
Dr. Juliane Heydenreich
juliane.heydenreich@uni-mainz.de

4 Supplementary Data: Characteristics of articles included in the systematic review.

Study	Design	*n* (sex)	Discipline, level	Age (yrs)	Ethnicity, country	Exercise	Environmental conditions	Interventions[1]	Outcomes[2]										
									Performance			Physiological					Perceptional		
									TT	TTE	MPO	SR	HR	BLa	T_c	T_{sk}	RPE	TS	TC
Aldous et al. 2019	Crossover, counterbalanced	8 (M)	Soccer, university-level (VO_2max 56 ± 9 ml · kg^{-1} · min^{-1})	22 ± 3	NR, UK	2 x 45-min INT Soccer Performance Test (in between 15 min half-time)	Chamber; 30.7 ± 0.3°C, 50.9 ± 4.2% RH	Pre- + mid-exercise: ingestion of non-CHO drink at -1°C (ICE) or room temperature (TN) within 30 min before exercise (7.5 g/kg) and at half-time (3.75 g/kg) in 3 serial aliquots				(✓)	(✓)		✓	✓	✓	✓	
Alhadad et al. 2021	Crossover, counterbalanced	10 (M)	NR, physically active (VO_2max 52 ± 6 ml · kg^{-1} · min^{-1})	24 ± 1	NR, Singapore	75 min running SS at 40% or 70% VO_2max	Laboratory; 25.1 ± 0.6°C, 63 ± 5% RH	Mid-exercise: ingestion of sports drink at ~-2°C (ICE) or ~26°C (TN) at 15 min intervals (5 x 2 g/kg) - sports drink contained 6.2% CHO				✓	✓		✓	✓	✓	✓	
Bain et al. 2012	Crossover, counterbalanced	9 (M)	NR, NR (VO_2peak 53.4 ± 3.6 ml · kg^{-1} · min^{-1})	22 ± 2	Caucasian, Canada	75 min cycling SS at 50% VO_2peak	Laboratory; 23.6 ± 0.6°C, 23 ± 11% RH	Pre- + mid-exercise: ingestion of water at 1.5°C (ICE), 10°C (COLD), 37°C (TN), or 50°C (WARM) 5 min before SS, and after 15, 30 and 45 min of SS in serial aliquots (4 x 3.2 ml/kg)				(✓)	✓		✓	✓			
Brade et al. 2014	Crossover, counterbalanced	12 (M)	Team sport players, NR	21.8 ± 2.3	NR, Australia	2 x 30 min sprint cycling (in between 10 min half-time)	Climate chamber; 35.2 ± 0.3°C, 57.8 ± 1.2% RH	Pre- + mid-exercise: ingestion of water at 0.6°C (ICE) or ~23°C (TN) within 30 min before exercise (7 g/kg) and at half-time (2.1 g/kg) in 3 serial aliquots			✓	✓	(✓)		✓	✓	(✓)	(✓)	
Burdon et al. 2010	Crossover, randomized	7 (M)	Cyclists, regional level (VO_2peak 59.4 ± 6.6 ml · kg^{-1} · min^{-1})	32.8 ± 6.1	NR, Australia	(1) 90 min cycling SS at 65% VO_2max, (2) 15 min cycling TT	Climate chamber; 28°C, 70% RH	Mid-exercise: ingestion of sports drink at (1) 4°C (COLD), (2) 37°C (TN), or (3) 37 °C + INT ingestion of ICE (-1°C; 30 mL; every 5 min), in serial aliquots during SS (9 x 2.3 mL/kg) - isocaloric intake in all trials - sports drink contained 7.4% CHO			✓	(✓)	(✓)		✓	✓	(✓)		(✓)
Burdon et al. 2013	Crossover, counterbalanced	10 (M)	Cyclists, NR (VO_2max 61.8 ± 5.6 ml · kg^{-1} · min^{-1})	30.1 ± 7.0	NR, Australia	(1) 90 min cycling SS at 60% VO_2peak, (2) 4 kJ/kg cycling TT	Climate chamber; 32°C, 40% RH	Mid-exercise: ingestion of sports drink at -1°C (ICE), 37°C (TN), or 37°C plus ICE MR (20 s, 25 g, every 5 min; WASH) during SS in serial aliquots (6 x 3.5 g/kg) - sports drink contained 7.4% CHO	✓		✓	(✓)	(✓)		(✓)	(✓)	(✓)		(✓)
Burdon et al. 2015	Crossover, randomized	10 (M)	Cyclists/triathletes, NR (VO_2max 61.8 ± 5.6 ml · kg^{-1} · min^{-1})	30.1 ± 7.0	NR, Australia	90 min cycling SS at 60% VO_2max	Climate chamber; 32°C, 40% RH	Mid-exercise: ingestion of sports drink at -1°C (ICE) or 37°C (TN) every 15 min of SS (6 x 3.5 mL/kg) - sports drink contained 7.4% CHO					(✓)		(✓)	✓			
Byrne et al. 2011	Crossover, randomized	7 (M)	Cyclists, recreational	21 ± 1.5	NR, UK	30 min cycling TT	Environmental chamber; 33	35-min pre-exercise: ingestion of 900 mL non-CHO sports drink at 37°C (TN) or 2°C	(✓)		✓	✓	✓	✓	✓	✓	✓		✓

							± 2°C, 61 ± 13% RH	(ICE) at 35, 25, and 10 min before exercise in serial aliquots (3 x 300 mL)											
Flood et al. 2017	Crossover, randomized, single-blind	8 (M)	NR, non-acclimated/fit (VO_2max 55.4 ± 6.0 ml · kg^{-1} · min^{-1})	26 ± 5	NR, UK	(1) cycling TTE at RPE = 16, (2) before and after TTE isokinetic cycling sprints	Heat chamber; 35.0 ± 0.8°C, 47.8 ± 2.3% RH	Pre- + mid-exercise: MR (MR temp ~19.7°C; each 25 mL; 5 s) before fixed RPE protocol and at 10-min intervals during TTE with MEN or PLA<br>- MEN: L-menthol solution (0.01%)<br>- PLA: apple-flavored non-calorific artificial sweetened		✓	✓	✓	(✓)		(✓)	(✓)		(✓)	(✓)
Gavel et al. 2021	Crossover, randomized	9 (F)	Cyclists, regional level (VO_2max 50.8 ± 6.0 ml · kg^{-1} · min^{-1})	26.7 ± 1.4	NR, Canada	(1) 30 km cycling TT, (2) before and after TT handgrip strength and maximal sprint tests	Environmental chamber; 30 ± 0.6 °C, 70 ± 1% RH	Mid-exercise:<br>MR at 7 times (MR temp 22°C; each 25 mL) during TT with PLA or MEN<br>- MEN: L-menthol solution (0.01%)<br>- PLA: non-caloric berry-flavored sweetener	✓		✓	✓	✓		(✓)		✓	✓	✓
Gerrett et al. 2017	Crossover, counterbalanced	12 (M)	NR, moderately to well-trained (VO_2max 58.5 ± 8.1 ml · kg^{-1} · min^{-1})	30.4 ± 3.4	NR, UK	31 min INT running	Climate-controlled room; 30.9 ± 0.9°C, 41.1 ± 4.0% RH	30-min pre-exercise: ingestion of 7.5 g/kg drink at 0.1 ± 0.1 °C (ICE) or 23.4 ± 0.9 °C (TN) in serial aliquots (3 x 2.5 g/kg)<br>- drinks contained 0.23% CHO				✓	✓	✓	✓	✓	✓	✓	
Gibson et al. 2019	Crossover, randomized	14 (11 M + 3 F)	Team sports players, trained (VO_2max 46.2 ± 12.9 ml · kg^{-1} · min^{-1})	24 ± 3	NR, UK	40 min INT cycling sprint protocol	Laboratory; 40°C, 50% RH	Mid-exercise: MR at 4 times (MR temp. 40°C; each 25 mL; 5 s) during exercise with MEN, water, or PLA<br>- MEN: L-menthol solution (0.01%)<br>- PLA: orange-flavored fruit squash (0.5% CHO)			✓	✓	✓		✓	✓	✓	✓	✓
Hailes et al. 2016	Crossover, randomized	12 (M)	NR, recreationally active (VO_2peak 61.5 ± 7.9 ml · kg^{-1} · min^{-1})	24 ± 4	NR, USA	3 h walking SS at 40% VO_2peak	Laboratory; 35.5°C, 50% RH	Mid-exercise: ingestion of water at 35.5°C (TN) or 0°C (ICE) during SS in serial aliquots (2 g/kg every 10 min)				✓	✓		✓	✓	✓		
Hue et al. 2013	Crossover, randomized	9 (5 M + 4 F)	Long-distance swimmers, internationally ranked	23.4 ± 3.3	NR, France	(1) 1000 m warm-up, (2) 10 x 100 m at competition pace, (3) 3000 m	Swimming pool; WBGT 27.5 ± 2.3°C, 73 ± 10% RH)	Mid-exercise: ingestion of 950 mL water at 1.3 ± 0.3°C (ICE) or 26.5 ± 2.5°C (TN) in serial aliquots (5 x 190 mL)				(✓)	✓		✓			✓	✓
Hue et al. 2015	Crossover, randomized	8 (5 M, 3 F)	Long-course swimmers, internationally ranked	24.4 ± 3.6	NR, France	5 km swimming SS at competition pace	Open water (WBGT: ~29.3°C)	Mid-exercise: ingestion of 950 mL water at 1.1 ± 0.7°C (ICE) or 28.0 ± 3.0°C (TN) in serial aliquots (5 x 190 mL)				✓	✓		✓			✓	✓
Ihsan et al. 2010	Crossover, counterbalanced	7 (M)	Cyclists/triathletes, trained	27.7 ± 3.1	NR, Australia	~40 km cycling TT (1200 kJ)	Climate chamber;30°C, 75% RH	30-min pre-exercise: ingestion of 6.8 g/kg water at 1.4 ± 1.1°C (ICE) or 26.8 ± 1.3°C (TN) in serial aliquots (150-200 g at 8-10 min intervals)	✓		✓	✓	(✓)	(✓)	(✓)	(✓)	(✓)	(✓)	

Iwata et al. 2020	Crossover, counterbalanced	24 (12 M, 12 F)	NR, healthy (VO_2max: M: 43.6 ± 3.3; F: 36.5 ± 4.2 ml · kg^{-1} · min^{-1})	M: 25.2± 1.7 F: 22.4 ± 1.5	NR, Japan	Cycling TTE at 55% VO_2max	Climate chamber; 38°C, 50% RH	30-min pre-exercise: ingestion of 7.5 g/kg sports drink at -1°C (ICE) or 20°C (TN) in serial aliquots (6 x 1.25 g/kg)<br>- sports drink contained 5.9% CHO	✓		✓	✓		✓	✓	✓	✓	✓
James et al. 2015	Crosssover, randomized	12 (M)	Runners, recreational (VO_2max 57.5 ± 4 ml · kg^{-1} · min^{-1})	38 ± 11	NR, UK	2 running GXT	Environmental chamber; 31.9 ± 1.0°C, 61 ± 8.9% RH	20-min pre-exercise: ingestion of 7.5 g/kg sports drink at -1°C (ICE) or 21°C (TN) in serial aliquots (4 x 1.88 g/kg)<br>- sports drink contained ~2.4 g CHO/100 mL			✓	✓	✓	✓	✓	✓	✓	
Jeffries et al. 2018	Crosssover, randomized, single-blind	10 (M)	NR, endurance trained (VO_2peak 52.4 ± 5.3 ml · kg^{-1} · min^{-1})	33 ± 9	NR, UK	Cycling TTE at 70% Wmax	Environmental chamber; 35 ± 0.2°C, 40 ± 0.5% RH	Mid-exercise:<br>MR at 1 time at 85% of TTE (25 mL; 5 s) with MEN or PLA<br>- MEN: L-menthol solution (0.01%)<br>- PLA: neutral, raspberry flavor, non-calorific solution	✓		(✓)	(✓)	(✓)	✓	(✓)	(✓)	(✓)	(✓)
Lamarche et al. 2015	Crosssover, randomized	10 (M)	NR, NR (VO_2peak 47.9 ± 9.8 ml · kg^{-1} · min^{-1})	25 ± 4	NR, Canada	75 min cycling SS at 50% VO_2peak	Calorimetric chamber; 25°C, ~25% RH	Pre- + mid-exercise: ingestion of water at 1.5°C (ICE) or 50°C (WARM) 5 min before SS, and after 15, 30, and 45 min of SS in serial aliquots (4 x 3.2 mL/kg)						✓	✓			
Lee & Shirreffs 2007	Crosssover, counterbalanced	9 (M)	NR, recreational (VO_2peak 50.0 ± 5.3 ml · kg^{-1} · min^{-1})	26 ± 6	NR, UK	(1) 90 min cycling SS at 50% VO_2peak, (2) cycling TTE at 95% VO_2peak	Laboratory; 25°C, 60% RH	Mid-exercise: ingestion of 1 L non-CHO beverage at 10°C (COLD), 37°C (TN) or 50°C (WARM) between 30 and 40 min in SS in serial aliquots (4 x 250 mL)	✓		✓	✓		✓	✓	(✓)	(✓)	
Lee et al. 2008a	Crosssover, counterbalanced	8 (M)	NR, moderately active (VO_2peak 53.8 ± 6.2 ml · kg^{-1} · min^{-1})	27 ± 4	NR, UK	(1) 90 min cycling SS at 50% VO_2peak, (2) cycling TTE at 95% VO_2peak	Laboratory; 25.4°C, 60% RH	Mid-exercise: ingestion of 1.6 L non-CHO drink at 10°C (COLD), 37°C (TN) or 50°C (WARM) at 30, 45, 60, and 75 min of SS (4 x 400 mL)	✓		✓	✓		✓	✓	(✓)	(✓)	
Lee et al. 2008b	Crosssover, counterbalanced	8 (M)	NR, recreational (VO_2peak 57.8 ± 5.6 ml · kg^{-1} · min^{-1})	22 ± 4	NR, UK	Cycling TTE at 65% VO_2peak	Environmental chamber; 35°C, 60% RH	Pre- + mid-exercise: ingestion of non-CHO drink at 4°C (COLD) or 37°C (TN) in serial aliquots within 30 min before (3 x 300 mL) and during TTE (100 mL every 10 min)	✓		✓			✓	✓	(✓)	(✓)	
Morris et al. 2014	Crosssover, counterbalanced	12 (M)	NR, NR (VO_2peak 53.9 ± 5.4 ml · kg^{-1} · min^{-1})	23 ± 3	NR, Canada	75 min cycling SS at 50% VO_2peak	Laboratory; 23.7 ± 1.3°C, 32 ± 10%	Pre- + mid-exercise: ingestion of water at 1.5°C (ICE), 37°C (TN), and 50°C (WARM) 5 min before SS, and after 15, 30 and 45 min of SS in serial aliquots (4 x 3.2 mL/kg)			✓			(✓)	(✓)			
Morris et al. 2016	Crosssover, counterbalanced	9 (M)	NR, healthy (VO_2peak 50.9 ± 8.5 ml · kg^{-1} · min^{-1})	25 ± 5	NR, Canada	75 min cycling SS at 55% VO_2peak	Laboratory; 33.5±1.4°C, 23.7±2.6% RH	Mid-exercise: ingestion of water at 37°C (TN) or ICE (1:2 mixture of shaved ice and 1.5°C water) in serial aliquots (3 x 3.2 mL/kg) in the first 45 min of SS			(✓)	✓		(✓)	(✓)			

Naito et al. 2020	Crosssover, randomized	7 (M)	NR, physically active	31 ± 4	NR, Japan	2 x 30 sets INT cycling sprint exercise (1 set = (1) 5 s max pedaling at the load of weight × 0.075 (kp), (2) 25 s of pedaling with no-workload, (3) 30 s of rest)	Climate chamber; 36.5 ± 0.5°C, 50 ± 3% RH	Mid-exercise: ingestion of 1.25 g/kg sports drink at -1°C (ICE) or 36.5°C (TN) at each break and 7.5 g/kg at the half-time<br>- sports drink contained 5.9% CHO		✓	✓	✓			✓	✓	✓	✓
Nakamura et al. 2020	Crosssover, randomized	8 (M)	NR, recreational (VO_2max 42.4 ml · kg^{-1} · min^{-1})	22 ± 1.3	NR, Japan	Cycling TTE at 75% VO_2max	Climate chamber; 35.0 ± 0.5°C, 62.9 ± 2.6% RH	15-min pre-exercise: ingestion 4 g/kg sports drink at -1°C (ICE) or room temperature (TN)<br>- sports drink contained 5.9% CHO	✓		✓	✓		✓	✓	✓	✓	✓
Ng et al. 2018	Crosssover, counterbalanced	8 (M)	NR, moderately - vigorous active	21 ± 4	NR, USA	30 min walking SS at 4 km/h and 12% incline wearing firefighter protective clothing	Laboratory; 35.2 ± 0.4°C, 39 ± 4% RH	Mid-exercise: ingestion of sports drink at -1.3 ± 0.2°C (ICE), 7.1 ± 1.5°C (COLD) or 22.4 ± 1.7°C (TN) in serial aliquots (12 x 1.25 g/kg)<br>- sports drink contained 5% CHO			✓	(✓)		(✓)	(✓)	✓	✓	
Ng et al. 2019	Crosssover, counterbalanced	8 (M)	NR, moderately - vigorous active (VO_2max 52.2 ± 7.9 ml · kg^{-1} · min^{-1})	22 ± 4	NR, USA	(1) 45 min cycling SS at 60% VO_2max, (2) cycling GXT	Environmental chamber; 35°C, 40% RH	Mid-exercise: ingestion of sports drink at-1.1 ± 0.5°C (ICE) or 22.8 ± 0.3°C (TN) during SS in serial aliquots (amount: individual sweat rate + 500 g; divided into 6 aliquots)<br>- sports drink contained CHO (amount NR)	✓	✓	✓	✓	✓	✓	✓	✓		
Onitsuka et al. 2020	Crosssover, counterbalanced	11 (M)	Healthy, NR (VO_2max 46.5 ± 9.8 ml · kg^{-1} · min^{-1})	22 ± 2	NR, Japan	60 min cycling SS at 50% VO_2max	Laboratory; 34°C	2 analyses:<br>(1) 30-min pre-exercise: ingestion of sports drink at 37°C (TN) or -1°C (ICE) in serial aliquots (5 x 1.5 g/kg)<br>(2) Pre- + mid-exercise: ingestion of sports drink at 37°C (TN) or -1°C (ICE) 30 min before exercise in serial aliquots (5 x 1.5 g/kg), and during SS in serial aliquots (6 x 1.25 g/kg)<br>- sports drink contained 6.2% CHO			✓	✓		✓	✓	✓	✓	✓
Parton et al. 2021	Crossover, randomized, double-blind	22 (11 F + 11 M)	NR, regular physical active (VO_2max: F: 43.5 ± 2.9; M: 53.9 ± 6.9 ml · kg^{-1} · min^{-1})	F: 22 ± 2 M: 20 ± 1	NR, UK	Cycling TTE at RPE of 16	Heat chamber; 34.9 ± 0.5°C, 40.6 ± 2.2 % RH	Pre- + mid-exercise: MR before TTE and every 10 min during TTE (MR temp. ~32°C; each 25 mL; 10 s) with MEN or CON<br>- MEN: L-menthol solution (0.01%)<br>- CON: apple flavored, non-calorific artificial sweetener	✓	✓	(✓)	(✓)		(✓)			(✓)	(✓)
Pryor et al. 2015	Crosssover, counterbalanced	10 (M)	Healthy, NR (VO_2max 50.5 ± 8.1 ml · kg^{-1} · min^{-1})	32.1 ± 8.3	NR, USA	45 min walking SS at 6.4 km/h wearing firefighting	Laboratory; ~39°C, ~17% RH	30-min pre-exercise: ingestion of 7.5 g/kg sports drink at 0.1°C (ICE) or 20°C (TN) in serial aliquots (6 x 1.25 g/kg)<br>- sports drink contained 3% CHO			✓	(✓)		(✓)	(✓)	(✓)	(✓)	(✓)

						equipment (~20.4 kg)													
Saldaris et al. 2020	Crosssover, counterbalanced	12 (M)	Long-distance runners, NR (VO_2max 61.1 ± 7.3 ml · kg^{-1} · min^{-1})	25.3 ± 4.2	NR, Australia	(1) 3 x 30 min running SS at 65% VO_2peak (before and in between resting periods with cognitive tests)<br>(2) running TTE at 100% VO_2peak	Climate chamber; 35.3 ± 0.3°C, 59.2 ± 2.5% RH	Pre- + mid-exercise: MR before and at the 15- and 30-min point of each block of SS and before TTE (each 25 mL; 5 s) with MEN or PLA<br>- MEN: menthol solution (0.1%) at 33.4 ± 0.5°C<br>PLA: water at 33.6 ± 0.7°C		(✓)		✓	✓		(✓)	(✓)	✓	✓	
Schulze et al. 2015	Crosssover, randomized	7 (M)	Triathletes, well trained (VO_2peak 61.7 ± 3.0 ml · kg^{-1} · min^{-1})	33 ± 8	NR, New Zealand	(1) 60 min cycling SS at RPE of 14, (2) 20 km cycling TT	Environmental chamber; 30°C, 80% RH	Mid-exercise: ingestion of sports drink at -1°C (ICE) or 37°C (TN) during SS in serial aliquots (2 x 7.5 g/kg)<br>- sports drink contained 6.2% CHO	✓		✓	✓	✓		✓	✓	✓	✓	✓
Siegel et al. 2011	Crosssover, counterbalanced	10 (M)	NR, recreational (VO_2peak 49.8 ± 4.7 ml · kg^{-1} · min^{-1})	24 ± 3	NR, Australia	(1) Running TTE at first ventilatory threshold, (2) before and after TTE 2-min sustained isometric MVC test	Laboratory; 34.1 ± 0.1°C, 49.5 ± 3.6% RH	Mid-exercise: ingestion of 1.25 g/kg sports drink at -1°C (ICE) or 40°C (WARM) after TTE<br>- sports drink contained 5% CHO		✓			✓		✓	✓			
Siegel et al. 2012	Crosssover, randomized	8 (M)	NR, moderately active (VO_2peak 54.2 ± 2.5 ml · kg^{-1} · min^{-1})	26 ± 4	NR, Australia	Running TTE at first ventilatory threshold	Climate chamber; 34°C, 52% RH	30-min pre-exercise: (1) ingestion of 7.5 g/kg sports drink at -1°C (ICE) or 37°C (TN) in serial aliquots (6 x 1.25 g/kg)<br>- sports drink contained 5% CHO		✓		✓	(✓)		✓	(✓)	(✓)	(✓)	
Snipe & Costa 2018	Crosssover, counterbalanced	6 (M), 6 (F)	Runners, trained (VO_2max 56 ± 6 ml · kg^{-1} · min^{-1})	37 ± 8	NR, Australia	120 min running SS at 60% VO_2max	Environmental chamber; 35.1 ± 0.5°C, 25 ± 3% RH	Pre- + mid-exercise: ingestion of water at 0.4 ± 0.4°C (ICE), 7.3 ± 0.8°C (COLD), or 22.1 ± 1.2°C (TN) immediately before, and every 15 min during SS in serial aliquots (8 x 3.75 mL/kg)				✓	✓		✓		✓	✓	
Stanley et al. 2010	Crosssover, counterbalanced	10 (M)	Cyclists/triathletes, trained (VO_2max 60.0 ± 7.7 ml · kg^{-1} · min^{-1})	30 ± 5	NR, Australia	(1) 75 min cycling SS at 58 ± 6% PPO, (2) 50 min recovery, (3) cycling TT (total work = 75% PPO x 30 min)	Climate chamber; 33.7 ± 0.8°C, 60.3 ± 2.0% RH	Mid-exercise: ingestion of 400 mL sports drink (5th minute) and each 200 mL (15th min, 25th min, 35th min) at 18.4 ± 0.5°C (TN) or -0.8 ± 0.1°C (ICE) during recovery<br>- sports drink contained 5.7 % CHO	✓		✓	✓	✓	✓	✓				
Stevens et al. 2016	Crosssover, randomized	11 (M)	Runners, moderately trained	29 ± 9	NR, Australia	5 km running TT	Environmental chamber; 32.6 ± 0.2 °C,	2 analyses:<br>(1) Mid-exercise:<br>MEN MR (MR temp. 22°C; each 25 mL; 5 s) at the 0.2 km mark of every 1 km	✓					(✓)	✓	(✓)	(✓)	(✓)	

							45.8 ± 5.7% RH	- MEN: L-menthol solution (0.01%)<br>- CON: no MR<br>(2) 35 - 5 min pre-exercise: ingestion of 7.5 g/kg sports drink at -1°C (ICE) or 22°C (TN) in serial aliquots (2 x 3.75 g/kg)<br>- sports drink contained 6% CHO											
Tabuchi et al. 2021	Crosssover, randomized	12 (M)	Firefighters, NR	24.4 ± 4.3	NR, Japan	(1) 10 min cycling SS at 125 W, (2) 20 min cycling SS at 75 W	Climate chamber; 35 °C, 50% RH	8 min pre-exercise: ingestion of 5 g/kg sports drink at 25°C (TN) and -1.7°C (ICE) in serial aliquots (2 x 2.5 g/kg)<br>- sports drink contained 6.2% CHO				✓	✓		✓		✓	✓	✓
Takeshima et al. 2017	Crosssover, counterbalanced	10 (M)	NR, active (PPO: 242 ± 27 W)	20.3 ± 1.6	NR, Japan	Cycling TTE at 55% PPO	Climate chamber;29.7 °C, 78.8% RH	0 – 15 min pre-exercise: ingestion of 7.5 g/kg sports drink at 37°C (TN) or -1°C (ICE) in serial aliquots (3 x 2.5 g/kg)<br>- 3 conditions: (1) ICE before and CON after warm-up, (2) CON before and ICE after warm-up, (3) CON before and after warm-up<br>- sports drink contained 5.5% CHO		✓		✓	✓		✓	✓	✓	✓	
Tay et al. 2016	Crosssover, counterbalanced	16 (M)	Military personnel, fit (estimated VO_2max 52 ± 3.3 ml · kg^{-1} · min^{-1})	21.8 ± 1.2	NR, Singapore	2 x 4 km walking SS at 5.3 km/h with 30 kg load (in between 15 min rest period)	Environmental chamber; 32°C, 70% RH	Pre- + mid-exercise: ingestion of water at 29°C (TN) or ice-slurry (ICE) 10 min before exercise, at 15 and 30 min of each work cycle, and during rest period in serial aliquots (6 x 200 mL)					✓		✓		✓		
Thomas et al. 2019	Crosssover, counterbalanced	10 (M)	NR, level 3 athletes (VO_2max 56.2 ± 6.6 ml · kg^{-1} · min^{-1})	30.5 ± 5.8	NR, UK	46 min INT running	Laboratory; 34.4 ± 1.4 °C, 36.3 ± 4.6 % RH	30-min pre-exercise: ingestion of 7.5 g/kg sports drink at 23.4 ± 0.2°C (TN) or -0.5 ± 0.4°C (ICE) in serial aliquots (3 x 2.5 g/kg)<br>- sports drink contained 0.75 g/kg CHO				✓	✓	✓	✓	✓	✓	✓	
Watkins et al. 2018	Crosssover, randomited	11 (M)	NR, physically active	20 ± 2	NR, UK	45 min INT walking exercise wearing protective clothing (~17 kg)	Environmental chamber; 49.6 ± 0.8°C; 15.4 ± 1.2% RH	15-min pre-exercise: ingestion of 500 mL (6.75 ± 0.84 g/kg) beverage at -1°C (ICE) or 22°C (TN)<br>- beverage contained 0.6% CHO				✓	✓		✓	✓	✓	✓	
Zimmermann & Landers 2015	Crosssover, randomited	9 (F)	Team sport athletes, trained	21 ± 1.2	NR, Australia	2 x 36 min INT cycling sprint protocol (in between 6 min recovery)	Climate chamber; 33.1 ± 0.1°C, 60.3 ± 1.5% RH	30-min pre-exercise: ingestion of 6.8 g/kg water at 25°C (TN) or -0.5°C (ICE) in serial aliquots (150-200 g servings)			✓		✓		✓		✓	✓	
Zimmermann et al. 2017a	Crosssover, randomited	10 (F)	Cyclists/triathletes, NR	28 ± 6	NR, Australia	800 kJ cycling TT	Environmental chamber; 34.9 ± 0.3°C, 49.8 ± 3.5% RH	30-min pre-exercise: ingestion of 7 g/kg of water at 0.5 ± 0.5°C (ICE) or 22.0 ± 2.0°C (TN) in serial aliquots (each 200 g at consistent time points)	✓		✓	✓	✓		✓	✓	✓	✓	

Zimmermann et al. 2017b	Crosssover, counterbalanced	10 (M)	NR, active (VO_2peak 48.5 ± 3.6 ml · kg^{-1} · min^{-1})	23 ± 3	NR, Australia	60 min cycling SS at 55 VO_2peak	Climate chamber; 35.0 ± 0.3°C, 50.2 ± 2.1% RH	<u>30-min pre-exercise:</u> ingestion of 7 g/kg water at ~0°C (ICE) or ~22°C (TN) in serial aliquots (each 200 g at consistent time points)	✓	✓		✓	✓	✓	✓

Note. [1] ICE: beverages with a temperature ≤ 2°C and studies stating that "ice-slurry" was ingested; COLD: beverages with a temperature >2 and ≤ 10°C; TN: beverages with a temperature >10 and ≤ 37°C and studies stating that "tepid" or "room temperature" drinks were ingested; WARM: beverages with a temperature > 37°C and ≤ 50°C. [2] ticks in brackets: quantitative data not reported. Abbreviations: ✓ = data could be used in meta-analysis, (✓) = data could not have been used for meta-analysis, F = female, BLa = blood lactate, CHO = carbohydrates, CON = control group, GXT = graded exercise test, HR = heart rate, INT = intermittent, M = male, MEN = menthol, MPO = mean power output, MR = mouth rinse, MVC = maximal voluntary contraction, NR = not reported, PLA = placebo group, RH = relative humidity, RPE = rate of perceived exertion, SR = sweat rate, T_c = core/rectal/gastrointestinal temperature, TC = thermal comfort, TS = thermal sensation, T_{sk} = skin temperature, TT = time trial, TTE = time to exhaustion, VO_2max = maximum oxygen consumption, WBGT = wet-bulb globe temperature; Wmax = maximum Watt

References

Aldous, J. W. F., Chrismas, B. C. R., Akubat, I., Stringer, C. A., Abt, G., & Taylor, L. (2019). Mixed-methods pre-match cooling improves simulated soccer performance in the heat. *European Journal of Sport Science*, *19*(2), Article 2. https://doi.org/10.1080/17461391.2018.1498542

Alhadad, S. B., Low, I. C. C., & Lee, J. K. W. (2021). Thermoregulatory responses to ice slurry ingestion during low and moderate intensity exercises with restrictive heat loss. *Journal of Science and Medicine in Sport*, *24*(1), 105–109. https://doi.org/10.1016/j.jsams.2020.07.002

Bain, A. R., Lesperance, N. C., & Jay, O. (2012). Body heat storage during physical activity is lower with hot fluid ingestion under conditions that permit full evaporation. *Acta Physiologica (Oxford, England)*, *206*(2), 98–108. https://doi.org/10.1111/j.1748-1716.2012.02452.x

Brade, C., Dawson, B., & Wallman, K. (2014). Effects of different precooling techniques on repeat sprint ability in team sport athletes. *European Journal of Sport Science*, *14*(sup1), S84–S91. https://doi.org/10.1080/17461391.2011.651491

Burdon, C. A., Hoon, M. W., Johnson, N. A., Chapman, P. G., & O'Connor, H. T. (2013). The effect of ice slushy ingestion and mouthwash on thermoregulation and endurance performance in the heat. *International Journal of Sport Nutrition and Exercise Metabolism*, *23*(5), 458–469. https://doi.org/10.1123/ijsnem.23.5.458

Burdon, C. A., Ruell, P., Johnson, N., Chapman, P., O'Brien, S., & O'Connor, H. T. (2015). The effect of ice-slushy consumption on plasma vasoactive intestinal peptide during prolonged exercise in the heat. *Journal of Thermal Biology*, *47*, 59–62. https://doi.org/10.1016/j.jtherbio.2014.11.005

Burdon, C., O'Connor, H., Gifford, J., Shirreffs, S., Chapman, P., & Johnson, N. (2010). Effect of drink temperature on core temperature and endurance cycling performance in warm, humid conditions. *Journal of Sports Sciences*, *28*(11), 1147–1156. https://doi.org/10.1080/02640414.2010.489197

Byrne, C., Owen, C., Cosnefroy, A., & Lee, J. K. W. (2011). Self-paced exercise performance in the heat after pre-exercise cold-fluid ingestion. *Journal of Athletic Training*, *46*(6), 592–599. https://doi.org/10.4085/1062-6050-46.6.592

Flood, T. R., Waldron, M., & Jeffries, O. (2017). Oral L-menthol reduces thermal sensation, increases work-rate and extends time to exhaustion, in the heat at a fixed rating of perceived exertion. *European Journal of Applied Physiology*, *117*(7), 1501–1512. https://doi.org/10.1007/s00421-017-3645-6

Gavel, E. H., Logan-Sprenger, H. M., Good, J., Jacobs, I., & Thomas, S. G. (2021). Menthol Mouth Rinsing and Cycling Performance in Females Under Heat Stress. *International Journal of Sports Physiology and Performance*, *16*(7), 1014–1020. https://doi.org/10.1123/ijspp.2020-0414

Gerrett, N., Jackson, S., Yates, J., & Thomas, G. (2017). Ice slurry ingestion does not enhance self-paced intermittent exercise in the heat. *Scandinavian Journal of Medicine & Science in Sports*, *27*(11), Article 11. https://doi.org/10.1111/sms.12744

Gibson, O. R., Wrightson, J. G., & Hayes, M. (2019). Intermittent sprint performance in the heat is not altered by augmenting thermal perception via L-menthol or capsaicin mouth rinses. *European Journal of Applied Physiology*, *119*(3), 653–664. https://doi.org/10.1007/s00421-018-4055-0

Hailes, W. S., Cuddy, J. S., Cochrane, K., & Ruby, B. C. (2016). Thermoregulation During Extended Exercise in the Heat: Comparisons of Fluid Volume and Temperature. *Wilderness & Environmental Medicine*, *27*(3), 386–392. https://doi.org/10.1016/j.wem.2016.06.004

Hue, O., Monjo, R., Lazzaro, M., Baillot, M., Hellard, P., Marlin, L., & Jean-Etienne, A. (2013). The effect of time of day on cold water ingestion by high-level swimmers in a tropical climate. *International Journal of Sports Physiology and Performance*, *8*(4), 442–451. https://doi.org/10.1123/ijspp.8.4.442

Hue, O., Monjo, R., & Riera, F. (2015). Imposed Cold-water Ingestion during Open Water Swimming in Internationally Ranked Swimmers. *International Journal of Sports Medicine*, *36*(11), 941–946. https://doi.org/10.1055/s-0035-1548812

Ihsan, M., Landers, G., Brearley, M., & Peeling, P. (2010). Beneficial effects of ice ingestion as a precooling strategy on 40-km cycling time-trial performance. *International Journal of Sports Physiology and Performance*, *5*(2), 140–151. https://doi.org/10.1123/ijspp.5.2.140

Iwata, R., Kawamura, T., Hosokawa, Y., Chang, L., Suzuki, K., & Muraoka, I. (2020). Differences between sexes in thermoregulatory responses and exercise time during endurance exercise in a hot environment following pre-cooling with ice slurry ingestion. *Journal of Thermal Biology*, *94*, 102746. https://doi.org/10.1016/j.jtherbio.2020.102746

James, C. A., Richardson, A. J., Watt, P. W., Gibson, O. R., & Maxwell, N. S. (2015). Physiological responses to incremental exercise in the heat following internal and external precooling. *Scandinavian Journal of Medicine & Science in Sports*, *25 Suppl 1*, 190–199. https://doi.org/10.1111/sms.12376

Jeffries, O., Goldsmith, M., & Waldron, M. (2018). L-Menthol mouth rinse or ice slurry ingestion during the latter stages of exercise in the heat provide a novel stimulus to enhance performance despite elevation in mean body temperature. *European Journal of Applied Physiology*, *118*(11), Article 11. https://doi.org/10.1007/s00421-018-3970-4

Lamarche, D. T., Meade, R. D., McGinn, R., Poirier, M. P., Friesen, B. J., & Kenny, G. P. (2015). Temperature of Ingested Water during Exercise Does Not Affect Body Heat Storage. *Medicine and Science in Sports and Exercise*, *47*(6), 1272–1280. https://doi.org/10.1249/MSS.0000000000000533

Lee, J. K. W., Maughan, R. J., & Shirreffs, S. M. (2008). The influence of serial feeding of drinks at different temperatures on thermoregulatory responses during cycling. *Journal of Sports Sciences*, *26*(6), 583–590. https://doi.org/10.1080/02640410701697388

Lee, J. K. W., & Shirreffs, S. M. (2007). The influence of drink temperature on thermoregulatory responses during prolonged exercise in a moderate environment. *Journal of Sports Sciences*, *25*(9), 975–985. https://doi.org/10.1080/02640410600959947

Lee, J. K. W., Shirreffs, S. M., & Maughan, R. J. (2008). Cold drink ingestion improves exercise endurance capacity in the heat. *Medicine and Science in Sports and Exercise*, *40*(9), 1637–1644. https://doi.org/10.1249/MSS.0b013e318178465d

Morris, N. B., Bain, A. R., Cramer, M. N., & Jay, O. (2014). Evidence that transient changes in sudomotor output with cold and warm fluid ingestion are independently modulated by abdominal, but not oral thermoreceptors. *Journal of Applied Physiology (Bethesda, Md.: 1985)*, *116*(8), 1088–1095. https://doi.org/10.1152/japplphysiol.01059.2013

Morris, N. B., Coombs, G., & Jay, O. (2016). Ice Slurry Ingestion Leads to a Lower Net Heat Loss during Exercise in the Heat. *Medicine & Science in Sports & Exercise*, *48*(1), Article 1. https://doi.org/10.1249/MSS.0000000000000746

Naito, T., Haramura, M., Muraishi, K., Yamazaki, M., & Takahashi, H. (2020). Impact of Ice Slurry Ingestion During Break-Times on Repeated-Sprint Exercise in the Heat. *Sports Medicine International Open*, *4*(02), Article 02. https://doi.org/10.1055/a-1139-1761

Nakamura, D., Muraishi, K., Hasegawa, H., Yasumatsu, M., & Takahashi, H. (2020). Effect of a cooling strategy combining forearm water immersion and a low dose of ice slurry ingestion on physiological response and subsequent exercise performance in the heat. *Journal of Thermal Biology*, *89*, 102530. https://doi.org/10.1016/j.jtherbio.2020.102530

Ng, J., Dobbs, W. C., & Wingo, J. E. (2019). Effect of Ice Slurry Ingestion on Cardiovascular Drift and V˙O2max during Heat Stress. *Medicine & Science in Sports & Exercise*, *51*(3), 582–589. https://doi.org/10.1249/MSS.0000000000001794

Ng, J., Wingo, J. E., Bishop, P. A., Casey, J. C., & Aldrich, E. K. (2018). Ice Slurry Ingestion and Physiological Strain During Exercise in Non-Compensable Heat Stress. *Aerospace Medicine and Human Performance*, *89*(5), 434–441. https://doi.org/10.3357/AMHP.4975.2018

Onitsuka, S., Zheng, X., & Hasegawa, H. (2020). Ice slurry ingestion before and during exercise inhibit the increase in core and deep-forehead temperatures in the second half of the exercise in a hot environment. *Journal of Thermal Biology*, *94*, 102760. https://doi.org/10.1016/j.jtherbio.2020.102760

Parton, A. J., Waldron, M., Clifford, T., & Jeffries, O. (2021). Thermo-behavioural responses to orally applied l-menthol exhibit sex-specific differences during exercise in a hot environment. *Physiology & Behavior*, *229*, 113250. https://doi.org/10.1016/j.physbeh.2020.113250

Pryor, R. R., Suyama, J., Guyette, F. X., Reis, S. E., & Hostler, D. (2015). The effects of ice slurry ingestion before exertion in Wildland firefighting gear. *Prehospital Emergency Care: Official Journal of the National Association of EMS Physicians and the National Association of State EMS Directors*, *19*(2), 241–246. https://doi.org/10.3109/10903127.2014.959221

Saldaris, J. M., Landers, G. J., & Lay, B. S. (2020). Physical and perceptual cooling: Improving cognitive function, mood disturbance and time to fatigue in the heat. *Scandinavian Journal of Medicine & Science in Sports*, *30*(4), Article 4. https://doi.org/10.1111/sms.13623

Schulze, E., Daanen, H. A. M., Levels, K., Casadio, J. R., Plews, D. J., Kilding, A. E., Siegel, R., & Laursen, P. B. (2015). Effect of thermal state and thermal comfort on cycling performance in the heat. *International Journal of Sports Physiology and Performance*, *10*(5), 655–663. https://doi.org/10.1123/ijspp.2014-0281

Siegel, R., Maté, J., Watson, G., Nosaka, K., & Laursen, P. B. (2011). The influence of ice slurry ingestion on maximal voluntary contraction following exercise-induced hyperthermia. *European Journal of Applied Physiology*, *111*(10), 2517–2524. https://doi.org/10.1007/s00421-011-1876-5

Siegel, R., Maté, J., Watson, G., Nosaka, K., & Laursen, P. B. (2012). Pre-cooling with ice slurry ingestion leads to similar run times to exhaustion in the heat as cold water immersion. *Journal of Sports Sciences*, *30*(2), 155–165. https://doi.org/10.1080/02640414.2011.625968

Snipe, R. M. J., & Costa, R. J. S. (2018). Does the temperature of water ingested during exertional-heat stress influence gastrointestinal injury, symptoms, and systemic inflammatory profile? *Journal of Science and Medicine in Sport*, *21*(8), 771–776. https://doi.org/10.1016/j.jsams.2017.12.014

Stanley, J., Leveritt, M., & Peake, J. M. (2010). Thermoregulatory responses to ice-slush beverage ingestion and exercise in the heat. *European Journal of Applied Physiology*, *110*(6), 1163–1173. https://doi.org/10.1007/s00421-010-1607-3

Stevens, C. J., Thoseby, B., Sculley, D. V., Callister, R., Taylor, L., & Dascombe, B. J. (2016). Running performance and thermal sensation in the heat are improved with menthol mouth rinse but not ice slurry ingestion: Running performance and thermal sensation. *Scandinavian Journal of Medicine & Science in Sports*, *26*(10), 1209–1216. https://doi.org/10.1111/sms.12555

Tabuchi, S., Horie, S., Kawanami, S., Inoue, D., Morizane, S., Inoue, J., Nagano, C., Sakurai, M., Serizawa, R., & Hamada, K. (2021). Efficacy of ice slurry and carbohydrate–electrolyte solutions for firefighters. *Journal of Occupational Health*, *63*(1). https://doi.org/10.1002/1348-9585.12263

Takeshima, K., Onitsuka, S., Xinyan, Z., & Hasegawa, H. (2017). Effect of the timing of ice slurry ingestion for precooling on endurance exercise capacity in a warm environment. *Journal of Thermal Biology*, *65*, 26–31. https://doi.org/10.1016/j.jtherbio.2017.01.010

Tay, C. S., Lee, J. K. W., Teo, Y. S., Q.Z. Foo, P., Tan, P. M. S., & Kong, P. W. (2016). Using gait parameters to detect fatigue and responses to ice slurry during prolonged load carriage. *Gait & Posture*, *43*, 17–23. https://doi.org/10.1016/j.gaitpost.2015.10.010

Thomas, G., Cullen, T., Davies, M., Hetherton, C., Duncan, B., & Gerrett, N. (2019). Independent or simultaneous lowering of core and skin temperature has no impact on self-paced intermittent running performance in hot conditions. *European Journal of Applied Physiology*, *119*(8), 1841–1853. https://doi.org/10.1007/s00421-019-04173-y

Watkins, E. R., Hayes, M., Watt, P., & Richardson, A. J. (2018). Practical pre-cooling methods for occupational heat exposure. *Applied Ergonomics*, *70*, 26–33. https://doi.org/10.1016/j.apergo.2018.01.011

Zimmermann, M., Landers, G. J., & Wallman, K. E. (2017). Crushed Ice Ingestion Does Not Improve Female Cycling Time Trial Performance in the Heat. *International Journal of Sport Nutrition and Exercise Metabolism*, *27*(1), 67–75. https://doi.org/10.1123/ijsnem.2016-0028

Zimmermann, M., Landers, G., Wallman, K. E., & Saldaris, J. (2017). The Effects of Crushed Ice Ingestion Prior to Steady State Exercise in the Heat. *International Journal of Sport Nutrition and Exercise Metabolism*, *27*(3), 220–227. https://doi.org/10.1123/ijsnem.2016-0215

Zimmermann, M. R., & Landers, G. J. (2015). The effect of ice ingestion on female athletes performing intermittent exercise in hot conditions. *European Journal of Sport Science*, *15*(5), 407–413. https://doi.org/10.1080/17461391.2014.965751
