# Supplementary material for: Effects of internal cooling on physical performance, physiological and perceptional parameters when exercising in the heat: A systematic review with meta-analyses
Source: Front Physiol. 2023 Apr 11;14:1125969. doi: 10.3389/fphys.2023.1125969 (PMC10126464; doi:10.3389/fphys.2023.1125969)
Supplement: Supplementary file 3 [file DataSheet6.pdf]

Supplementary Material 6

Effects of internal cooling on physical performance, physiological and perceptional parameters when exercising in the heat: a systematic review with meta-analyses

Juliane Heydenreich*, Karsten Koehler, Hans Braun, Mareike Grosshauser, Helmut Heseker, Daniel Koenig, Alfonso Lampen, Stephanie Mosler, Andreas Niess, Alexandra Schek, Anja Carlsohn

*** Correspondence:**
Dr. Juliane Heydenreich
juliane.heydenreich@uni-mainz.de

6 **Supplementary Data:** Results of simple linear meta-regressions with dose, heat index, and exercise duration as independent variables, and SMD of performance, physiological and perceptional outcomes as dependent variables.

	Coefficients			Model Summary	
	B	**SE *B***	**β**	**R^2**	***p*-value**
**Time trial performance**					
Constant	0.22	0.31		.46	.09
Dose [g/kg]	-0.05	0.03	-.68		
Constant	-2.43	1.69		.23	.27
Heat index	0.06	0.05	.48		
Constant	0.39	0.45		.37	.15
Exercise duration [min]	-0.01	0.01	-.61		
**Time to exhaustion**					
Constant	0.49	0.46		.00	.87
Dose [g/kg]	0.01	0.03	.05		
Constant	-0.83	0.93		.17	.16
Heat index	0.04	0.03	.42		
Constant	1.16	0.55		.12	.25
Exercise duration [min]	-0.01	0.01	-.34		
**Mean Power Output**					
Constant	0.10	0.22		.05	.49
Dose [g/kg]	0.01	0.02	.21		
Constant	1.07	0.70		.11	.26
Heat index	-0.02	0.02	-.34		
Constant	0.03	0.3		.05	.47
Exercise duration [min]	0.00	0.00	.22		
**Core temperature**					
Constant	0.03	0.17		.05	.12

Dose [g/kg]	-0.02	0.01	-.23		
Constant	-0.46	0.42		.01	.53
Heat index	0.01	0.01	.09		
Constant	0.05	0.22		.04	.23
Exercise duration [min]	-0.00	0.00	-.19		
**Skin temperature**					
Constant	0.61	0.18		.43	**<0.001**
Dose [g/kg]	-0.06	0.01	-.66		
Constant	0.09	0.49		.01	.55
Heat index	-0.01	0.01	-.10		
Constant	0.79	0.23		.40	**<0.001**
Exercise duration [min]	-0.01	0.00	-.63		
**Sweat rate**					
Constant	-0.10	0.15		.03	.30
Dose [g/kg]	-0.01	0.01	-.17		
Constant	-0.91	0.38		.08	.08
Heat index	0.02	0.01	.28		
Constant	-0.20	0.18		.00	.75
Exercise duration [min]	-0.00	0.00	-.05		
**Heart rate**					
Constant	0.11	0.08		.28	**<.001**
Dose [g/kg]	-0.02	0.01	-.53		
Constant	-0.14	0.22		.00	.96
Heat index	0.00	0.01	-.01		
Constant	0.20	0.10		.28	**<0.01**
Exercise duration [min]	-0.01	0.00	-.53		
**Rate of perceived exertion**					
Constant	-0.13	0.11		.01	.56
Dose [g/kg]	-0.00	0.01	-.11		
Constant	0.12	0.39		.02	.44
Heat index	-0.01	0.01	-.15		
Constant	-0.14	0.13		.01	.68
Exercise duration [min]	-0.00	0.00	-.08		
**Thermal sensation**					
Constant	-0.24	0.11		.02	.49
Dose [g/kg]	0.01	0.01	.13		
Constant	-0.13	0.34		.00	.89
Heat index	0.00	0.01	-.03		
Constant	-0.28	0.13		.02	.51
Exercise duration [min]	0.00	0.00	.14		
**Thermal comfort**					
Constant	0.24	0.23		.12	.28
Dose [g/kg]	-0.02	0.02	-.34		
Constant	0.43	0.50		.07	.40
Heat index	-0.01	0.01	-.27		
Constant	0.30	0.24		.26	.16

Exercise duration [min]	-0.01	0.01	-.51

Note. *p*-values in bold demonstrate statistical significance.
