# Supplementary material for: Effects of internal cooling on physical performance, physiological and perceptional parameters when exercising in the heat: A systematic review with meta-analyses
Source: Front Physiol. 2023 Apr 11;14:1125969. doi: 10.3389/fphys.2023.1125969 (PMC10126464; doi:10.3389/fphys.2023.1125969)
Supplement: Supplementary file 4 [file DataSheet3.PDF]

Supplementary Material 3

Effects of internal cooling on physical performance, physiological and perceptional parameters when exercising in the heat: a systematic review with meta-analyses

Juliane Heydenreich*, Karsten Koehler, Hans Braun, Mareike Grosshauser, Helmut Heseker, Daniel Koenig, Alfonso Lampen, Stephanie Mosler, Andreas Niess, Alexandra Schek, Anja Carlsohn

*** Correspondence:**
Dr. Juliane Heydenreich
juliane.heydenreich@uni-mainz.de

3 Supplementary Data: List of excluded articles and reason for exclusion.

Reference	Reason for exclusion
Ashworth et al. 2021 (1)	Age not reported
Best et al. 2018 (2)	Inclusion criteria for subjects not met
Best et al. 2020 (3)	No quantitative data reported
Burdon et al. 2014 (4)	Inclusion criteria for outcomes not met
Chaseling et al. 2018 (5)	Inclusion criteria for subjects not met (MS patients)
De Carvalho et al. 2015 (6)	No isovolumic drink ingestion
Deshayes et al. 2019 (7)	Inclusion criteria for study type not met
Dugas 2011 (8)	Duplicate publication (Siegel et al. 2010)
Forsyth et al. 2016 (9)	No exercise performed
Fuchs et al. 2020 (10)	External cooling (water immersion)
Gillis et al. 2015 (11)	External cooling (menthol)
Gosselin et al. 2019 (12)	No control group
Hasegawa et al. 2006 (13)	Combination of internal and external cooling
Kazadi et al. 2018 (14)	Use of natural substances containing menthol (Peppermint oil); no exercise performed
Lee et al. 2013 (15)	Application of internal cooling after exercise (“post-cooling”)
Levels et al. 2013 (16)	No isocaloric drink ingestion
Li et al. 2018 (17)	Inclusion criteria for subjects not met (mice)
Maunder et al. 2017 (18)	No isocaloric intake
Meamarbashi 2014 (19)	Use of natural substances containing menthol (Peppermint essential oil)
Meamarbashi & Rajabi 2013 (20)	Use of natural substances containing menthol (Peppermint oil)
Mejuto et al. 2018 (21)	Combination of internal and external cooling
Morris et al. 2014 (22)	No oral intake (control group with nasogastric tube)

Morris et al. 2017 (23)	No exercise performed, cold environment
Mündel et al. 2006 (24)	No isovolumic drink ingestion
Mündel & Jones 2010 (25)	No isovolumic drink ingestion
Naito & Ogaki 2017 (26)	No quantitative data reported
Naito et al. 2017 (27)	No quantitative data reported
Naito et al. 2018 (28)	No quantitative data reported
Naito et al. 2021 (29)	Combination of internal and external cooling
Onitsuka et al. 2018 (30)	No exercise performed
Racinais et al. 2021 (31)	Inclusion criteria for study type not met
Riera et al. 2014 (32)	No quantitative data reported
Riera et al. 2016 (33)	No quantitative data reported
Ross et al. 2011 (34)	Combination of internal and external cooling
Ross et al. 2012 (35)	Combination of internal and external cooling
Saldaris et al. 2019 (36)	Duplicate data publication
Savoie et al. 2015 (37)	Combination of internal and external cooling; no control group
Scarfò et al. 2021 (38)	Inclusion criteria for subjects not met (not healthy)
Shepherd & Peart 2017 (39)	Use of natural substances containing menthol (Peppermint oil)
Siegel et al. 2010 (40)	No quantitative data reported
Sönmez et al. 2010 (41)	Use of natural substances containing menthol (Mint extract)
Stevens et al. 2013 (42)	No isovolumic drink ingestion
Stevens et al. 2017 (43)	Combination of internal and external cooling
Stevens et al. 2021 (44)	Age not reported
Stevens et al. 2020 (45)	Combination of internal and external cooling
Tasing et al. 2016 (46)	Inclusion criteria for subjects not met (obese men)
Trbovich 2019 (47)	Inclusion criteria for subjects not met
Trong et al. 2015 (48)	No quantitative data reported
Valente et al. 2015 (49)	No exercise performed
Walker et al. 2014 (50)	No isocaloric drink ingestion, post-cooling
Wimer 2009 (51)	No adequate control group
Xu et al. 2021 (52)	No isocaloric drink ingestion
Yeo et al. 2012 (53)	No isovolumic drink ingestion
Zhang et al. 2014 (54)	Combination of menthol and other substance (benzocaine)

References

1. Ashworth ET, Cotter JD, Kilding AE. Impact of elevated core temperature on cognition in hot environments within a military context. Eur J Appl Physiol. 2021 Apr;121(4):1061–71.

2. Best R, Spears I, Hurst P, Berger N. The Development of a Menthol Solution for Use during Sport and Exercise. Beverages. 2018 Jun 11;4(2):44.

3. Best R, Temm D, Hucker H, McDonald K. Repeated Menthol Mouth Swilling Affects Neither Strength nor Power Performance. Sports. 2020 Jun 17;8(6):90.

4. Burdon CA, Easthope CS, Johnson NA, Chapman PG, O'Connor H. The influence of ice slushy on voluntary contraction force following exercise-induced hyperthermia. Appl Physiol Nutr Metab. 2014 Jul;39(7):781–6.

5. Chaseling GK, Filingeri D, Barnett M, Hoang P, Davis SL, Jay O. Cold Water Ingestion Improves Exercise Tolerance of Heat-Sensitive People with MS. Med Sci Sports Exerc. 2018 Apr;50(4):643–8.

6. De Carvalho MV, De Andrade MT, Ramos GP, Maia-Lima A, Pereira ER, Mendes TT, et al. The temperature of water ingested ad libitum does not influence performance during a 40-km self-paced cycling trial in the heat. J Sports Med Phys Fitness. 2015 Dec;55(12):1473–9.

7. Deshayes TA, De La Flore A, Gosselin J, Beliveau J, Jeker D, Goulet EDB. The Impact of an Ice Slurry-Induced Gastrointestinal Heat Sink on Gastrointestinal and Rectal Temperatures Following Exercise. Sports. 2019 Aug 27;7(9):198.

8. Dugas J. Ice slurry ingestion increases running time in the heat. Clin J Sport Med. 2011 Nov;21(6):541–2.

9. Forsyth P, Pumpa K, Knight E, Miller J. Physiological and perceptual effects of precooling in wheelchair basketball athletes. J Spinal Cord Med. 2016 Nov;39(6):671–8.

10. Fuchs CJ, Kouw IWK, Churchward-Venne TA, Smeets JSJ, Senden JM, Lichtenbelt WD van M, et al. Postexercise cooling impairs muscle protein synthesis rates in recreational athletes. J Physiol. 2020 Feb;598(4):755–72.

11. Gillis DJ, Weston N, House JR, Tipton MJ. Influence of repeated daily menthol exposure on human temperature regulation and perception. Physiol Behav. 2015 Feb;139:511–8.

12. Gosselin J, Béliveau J, Hamel M, Casa D, Hosokawa Y, Morais JA, et al. Wireless measurement of rectal temperature during exercise: Comparing an ingestible thermometric telemetric pill used as a suppository against a conventional rectal probe. Journal of Thermal Biology. 2019 Jul;83:112–8.

13. Hasegawa H, Takatori T, Komura T, Yamasaki M. Combined effects of pre-cooling and water ingestion on thermoregulation and physical capacity during exercise in a hot environment. J Sports Sci. 2006 Jan;24(1):3–9.

14. Kazadi LC, Fletcher J, Barrow PA. Gastric cooling and menthol cause an increase in cardiac parasympathetic efferent activity in healthy adult human volunteers. Exp Physiol. 2018 Oct;103(10):1302–8.

15. Lee JKW, Yeo ZW, Nio AQX, Koh ACH, Teo YS, Goh LF, et al. Cold drink attenuates heat strain during work-rest cycles. Int J Sports Med. 2013 Dec;34(12):1037–42.

16. Levels K, Teunissen L PJ, de Haan A, de Koning JJ, van Os B, Daanen H AM. Effect of warm-up and precooling on pacing during a 15-km cycling time trial in the heat. Int J Sports Physiol Perform. 2013 May;8(3):307–11.

17. Li C, Li J, Xiong X, Liu Y, Lv Y, Qin S, et al. TRPM8 activation improves energy expenditure in skeletal muscle and exercise endurance in mice. Gene. 2018 Jan 30;641:111–6.

18. Maunder E, Laursen PB, Kilding AE. Effect of ad Libitum Ice-Slurry and Cold-Fluid Ingestion on Cycling Time-Trial Performance in the Heat. International Journal of Sports Physiology and Performance. 2017 Jan;12(1):99–105.

19. Meamarbashi A. Instant effects of peppermint essential oil on the physiological parameters and exercise performance. Avicenna J Phytomed. 2014 Jan;4(1):72–8.

20. Meamarbashi A, Rajabi A. The effects of peppermint on exercise performance. J Int Soc Sports Nutr. 2013 Mar 21;10(1):15.

21. Mejuto G, Chalmers S, Gilbert S, Bentley D. The effect of ice slurry ingestion on body temperature and cycling performance in competitive athletes. Journal of Thermal Biology. 2018 Feb;72:143–7.

22. Morris NB, Bain AR, Cramer MN, Jay O. Evidence that transient changes in sudomotor output with cold and warm fluid ingestion are independently modulated by abdominal, but not oral thermoreceptors. J Appl Physiol (1985). 2014 Apr 15;116(8):1088–95.

23. Morris NB, Filingeri D, Halaki M, Jay O. Evidence of viscerally-mediated cold-defence thermoeffector responses in man. J Physiol. 2017 Feb 15;595(4):1201–12.

24. Mündel T, King J, Collacott E, Jones DA. Drink temperature influences fluid intake and endurance capacity in men during exercise in a hot, dry environment. Exp Physiol. 2006 Sep;91(5):925–33.

25. Mündel T, Jones DA. The effects of swilling an L(-)-menthol solution during exercise in the heat. Eur J Appl Physiol. 2010 May;109(1):59–65.

26. Naito T, Ogaki T. Comparison of the effects of cold water and ice ingestion on endurance cycling capacity in the heat. J Sport Health Sci. 2017 Mar;6(1):111–7.

27. Naito T, Iribe Y, Ogaki T. Ice ingestion with a long rest interval increases the endurance exercise capacity and reduces the core temperature in the heat. J Physiol Anthropol. 2017 Dec;36(1):9.

28. Naito T, Sagayama H, Akazawa N, Haramura M, Tasaki M, Takahashi H. Ice slurry ingestion during break times attenuates the increase of core temperature in a simulation of physical demand of match-play tennis in the heat. Temperature. 2018 Oct 2;5(4):371–9.

29. Naito T, Nakamura M, Muraishi K, Eda N, Ando K, Takemura A, et al. In-play optimal cooling for outdoor match-play tennis in the heat. European Journal of Sport Science. 2021 Jan 18;1–10.

30. Onitsuka S, Nakamura D, Onishi T, Arimitsu T, Takahashi H, Hasegawa H. Ice slurry ingestion reduces human brain temperature measured using non-invasive magnetic resonance spectroscopy. Sci Rep. 2018 Feb 9;8(1):2757.

31. Racinais S, Ihsan M, Taylor L, Cardinale M, Adami PE, Alonso JM, et al. Hydration and cooling in elite athletes: relationship with performance, body mass loss and body temperatures during the Doha 2019 IAAF World Athletics Championships. Br J Sports Med. 2021 Feb 12;bjsports-2020-103613.

32. Riera F, Trong TT, Sinnapah S, Hue O. Physical and perceptual cooling with beverages to increase cycle performance in a tropical climate. PLoS One. 2014;9(8):e103718.

33. Riera F, Trong TT, Rinaldi K, Hue O. Precooling does not Enhance the Effect on Performance of Midcooling with Ice-Slush/Menthol. Int J Sports Med. 2016 Dec;37(13):1025–31.

34. Ross MLR, Garvican LA, Jeacocke NA, Laursen PB, Abbiss CR, Martin DT, et al. Novel precooling strategy enhances time trial cycling in the heat. Med Sci Sports Exerc. 2011 Jan;43(1):123–33.

35. Ross ML, Jeacocke NA, Laursen PB, Martin DT, Abbiss CR, Burke LM. Effects of lowering body temperature via hyperhydration, with and without glycerol ingestion and practical precooling on cycling time trial performance in hot and humid conditions. J Int Soc Sports Nutr. 2012 Dec 17;9(1):55.

36. Saldaris JM, Landers GJ, Lay BS, Zimmermann MR. Internal precooling decreases forehead and core temperature but does not alter choice reaction time during steady state exercise in hot, humid conditions. Journal of Thermal Biology. 2019 Apr;81:66–72.

37. Savoie FA, Dion T, Asselin A, Gariepy C, Boucher PM, Berrigan F, et al. Intestinal temperature does not reflect rectal temperature during prolonged, intense running with cold fluid ingestion. Physiol Meas. 2015 Feb;36(2):259–72.

38. Scarfò G, Fusi J, Franzoni F. Paroxysmal Atrial Fibrillation Induced by Ice-Cold Water Ingestion in a Triathlete: A Case Report. Am J Case Rep [Internet]. 2021 Jun 11 [cited 2022 Feb 22];22. Available from: https://www.amjcaserep.com/abstract/index/idArt/931460

39. Shepherd K, Peart DJ. Aerobic capacity is not improved following 10-day supplementation with peppermint essential oil. Appl Physiol Nutr Metab. 2017 May;42(5):558–61.

40. Siegel R, Maté J, Brearley MB, Watson G, Nosaka K, Laursen PB. Ice Slurry Ingestion Increases Core Temperature Capacity and Running Time in the Heat. Medicine & Science in Sports & Exercise. 2010 Apr;42(4):717–25.

41. Sönmez G, Çolak M, Sönmez S, Schoenfeld B. Effects of oral supplementation of mint extract on muscle pain and blood lactate. Biomedical Human Kinetics. 2010 Jan 1;2(2010):66–9.

42. Stevens CJ, Dascombe B, Boyko A, Sculley D, Callister R. Ice slurry ingestion during cycling improves Olympic distance triathlon performance in the heat. Journal of Sports Sciences. 2013 Aug;31(12):1271–9.

43. Stevens CJ, Bennett KJM, Sculley DV, Callister R, Taylor L, Dascombe BJ. A Comparison of Mixed-Method Cooling Interventions on Preloaded Running Performance in the Heat. J Strength Cond Res. 2017 Mar;31(3):620–9.

44. Stevens CJ, Ross MLR, Vogel RM. Development of a "Cooling" Menthol Energy Gel for Endurance Athletes: Effect of Menthol Concentration on Acceptability and Preferences. International Journal of Sport Nutrition and Exercise Metabolism. 2021 Jan 1;31(1):40–5.

45. Stevens CJ, Ross ML, Périard JD, Vallance BS, Burke LM. Core Temperature Responses to Elite Racewalking Competition. International Journal of Sports Physiology and Performance. 2020 Jul 1;15(6):892–5.

46. Tasing P, Kulaputana O, Sanguanrungsirikul S, Kaikaew K. Effects of Precooling with Cold Water Ingestion on Thermoregulatory Response of Obese Men during Moderate Intensity Exercise in Hot and Humid Environment. J Med Assoc Thai. 2016 Feb;99(2):197–205.

47. Trbovich M. Efficacy of Various Cooling Techniques During Exercise in Persons With Spinal Cord Injury: A Pilot Crossover Intervention Study. Topics in Spinal Cord Injury Rehabilitation. 2019 Jan;25(1):74–82.

48. Tran Trong T, Riera F, Rinaldi K, Briki W, Hue O. Ingestion of a Cold Temperature/Menthol Beverage Increases Outdoor Exercise Performance in a Hot, Humid Environment. Romanovsky AA, editor. PLoS ONE. 2015 Apr 9;10(4):e0123815.

49. Valente A, Carrillo AE, Tzatzarakis MN, Vakonaki E, Tsatsakis AM, Kenny GP, et al. The absorption and metabolism of a single L-menthol oral versus skin administration: Effects on thermogenesis and metabolic rate. Food Chem Toxicol. 2015 Dec;86:262–73.

50. Walker A, Driller M, Brearley M, Argus C, Rattray B. Cold-water immersion and iced-slush ingestion are effective at cooling firefighters following a simulated search and rescue task in a hot environment. Appl Physiol Nutr Metab. 2014 Oct;39(10):1159–66.

51. Wimer GS. Wearing a cap, thermoregulation, and thermal sensation during running in a hot environment. J Sports Med Phys Fitness. 2009 Sep;49(3):272–7.

52. Xu M, Wu Z, Dong Y, Qu C, Xu Y, Qin F, et al. A Mixed-Method Approach of Pre-Cooling Enhances High-Intensity Running Performance in the Heat. J Sports Sci Med. 2021 Mar;20(1):26–34.

53. Yeo Z, Fan PW, Nio AQ, Byrne C, Lee JK. Ice Slurry on Outdoor Running Performance in Heat. Int J Sports Med. 2012 Jun 22;33(11):859–66.

54. Zhang Y, Balilionis G, Casaru C, Geary C, Schumacker RE, Neggers YH, et al. Effects of caffeine and menthol on cognition and mood during simulated firefighting in the heat. Appl Ergon. 2014 May;45(3):510–4.
