# Supplementary material for: Effects of internal cooling on physical performance, physiological and perceptional parameters when exercising in the heat: A systematic review with meta-analyses
Source: Front Physiol. 2023 Apr 11;14:1125969. doi: 10.3389/fphys.2023.1125969 (PMC10126464; doi:10.3389/fphys.2023.1125969)
Supplement: Supplementary file 5 [file DataSheet1.PDF]

Supplementary Material 1

Effects of internal cooling on physical performance, physiological and perceptional parameters when exercising in the heat: a systematic review with meta-analyses

Juliane Heydenreich*, Karsten Koehler, Hans Braun, Mareike Grosshauser, Helmut Heseker, Daniel Koenig, Alfonso Lampen, Stephanie Mosler, Andreas Niess, Alexandra Schek, Anja Carlsohn

*** Correspondence:**
Dr. Juliane Heydenreich
juliane.heydenreich@uni-mainz.de

1 Supplementary Data: Details of search strategy.

Database	MEDLINE (via PubMed)
Date of search	Dec 17, 2021
Keywords	(athlet* OR active) AND ((("2015"[Date - Publication] : "3000"[Date - Publication])) AND ((performance OR "side effect*" OR "side-effect*" OR "core temperature" OR "skin temperature" OR "gastrointestinal temperature" OR "rectal temperature" OR lactate OR "heart rate" OR heart-rate OR "thermal sensation" OR "thermal comfort" OR "rate of perceived exertion") AND (ice-slush* OR "ice ingestion" OR ice-slurr* OR menthol OR "internal cooling" OR cold-water OR "cold water")))
Language	German OR English
Species	humans
