# Supplementary material for: Effects of internal cooling on physical performance, physiological and perceptional parameters when exercising in the heat: A systematic review with meta-analyses
Source: Front Physiol. 2023 Apr 11;14:1125969. doi: 10.3389/fphys.2023.1125969 (PMC10126464; doi:10.3389/fphys.2023.1125969)
Supplement: Supplementary file 6 [file DataSheet5.pdf]

frontiers

Supplementary Material 5

Effects of internal cooling on physical performance, physiological and perceptional parameters when exercising in the heat: a systematic review with meta-analyses

Juliane Heydenreich*, Karsten Koehler, Hans Braun, Mareike Grosshauser, Helmut Heseker, Daniel Koenig, Alfonso Lampen, Stephanie Mosler, Andreas Niess, Alexandra Schek, Anja Carlsohn

*** Correspondence:**
Dr. Juliane Heydenreich
juliane.heydenreich@uni-mainz.de

5 Supplementary Data: Risk of bias summary.

	Random sequence generation (selection bias)	Allocation concealment (selection bias)	Blinding of participants and personnel (performance bias)	Incomplete outcome data (attrition bias)	Selective reporting (reporting bias)	Other bias
Aldous et al. 2019	?	−	−	+	−	+
Alhadad et al. 2021	?	−	−	+	?	+
Bain et al. 2012	?	−	−	+	−	+
Brade et al. 2014	?	−	−	+	−	+
Burdon et al. 2010	?	−	−	+	−	+
Burdon et al. 2013	?	−	−	+	−	+
Burdon et al. 2015	?	−	−	+	−	+
Byrne et al. 2011	?	−	−	+	?	+
Flood et al. 2017	+	−	?	+	−	+
Gavel et al. 2021	?	−	−	+	−	+
Gerrett et al. 2017	?	−	−	+	?	+
Gibson et al. 2019	?	−	−	+	−	+
Hailes et al. 2016	?	−	−	+	?	+
Hue et al. 2013	?	−	−	+	−	+
Hue et al. 2015	?	−	−	+	?	+
Ihsan et al. 2010	?	−	−	+	−	+
Iwata et al. 2020	?	−	−	+	−	+
James et al. 2015	?	−	−	+	−	+
Jeffries et al. 2018	+	−	?	+	−	+
Lamarche et al. 2015	?	−	−	+	?	+
Lee & Shirreffs 2007	?	−	−	+	−	+
Lee at al. 2008b	?	−	−	+	−	+
Lee et al. 2008a	?	−	−	+	−	+
Morris et al. 2014	?	−	−	+	−	+
Morris et al. 2016	?	−	−	+	−	+
Naito et al. 2020	?	−	−	+	−	+
Nakamura et al. 2020	?	−	−	+	−	+
Ng et al. 2018	?	−	−	+	−	+
Ng et al. 2019	?	−	−	+	−	+
Onitsuka et al. 2020	?	−	−	+	−	+
Parton et al. 2021	?	−	+	+	−	+
Pryor et al. 2015	?	−	−	+	−	+
Saldaris et al. 2020	?	−	−	+	−	+
Schulze et al. 2015	?	−	−	+	−	+
Siegel et al. 2011	?	−	−	+	?	+
Siegel et al. 2012	?	−	−	+	−	+
Snipe & Costa 2018	?	−	−	+	−	+
Stanley et al. 2010	?	−	−	+	−	+
Stevens et al. 2016	?	−	−	+	−	+
Tabuchi et al. 2021	?	−	−	+	−	+
Takeshima et al. 2017	?	−	−	+	−	+
Tay et al. 2016	?	−	−	+	−	+
Thomas et al. 2019	?	−	−	+	−	+
Watkins et al. 2018	?	−	−	+	?	+
Zimmermann & Landers 2015	?	−	−	+	−	+
Zimmermann et al. 2017a	?	−	−	+	−	+
Zimmermann et al. 2017b	?	−	−	+	−	+
